# Systemic Treatment Strategies for Patients with Psoriasis and Psoriatic Arthritis in the Setting of ANA Positivity or Lupus Spectrum Disease: A Comprehensive Systematic Review

**DOI:** 10.3390/ijms27021093

**Published:** 2026-01-22

**Authors:** Jeng-Wei Tjiu, Tsen-Fang Tsai

**Affiliations:** Department of Dermatology, National Taiwan University Hospital and National Taiwan University College of Medicine, Taipei 100225, Taiwan

**Keywords:** psoriasis, psoriatic arthritis, cutaneous lupus erythematosus, systemic lupus erythematosus, antinuclear antibodies, biologic therapy, IL-23 inhibitors, IL-17 inhibitors, TNF inhibitors, TYK2 inhibition, drug-induced lupus, overlap autoimmune disease, immunodermatology, systemic therapy safety

## Abstract

Psoriasis and psoriatic arthritis (PsA) occasionally coexist with antinuclear antibody (ANA) positivity, cutaneous lupus erythematosus (CLE), or systemic lupus erythematosus (SLE), creating one of the most challenging therapeutic overlap scenarios in immunodermatology. Divergent immune pathways—IL-23/Th17-driven psoriatic inflammation versus type I interferon-mediated autoimmunity—generate unique vulnerabilities when systemic treatments are used. To synthesize treatment outcomes, lupus-related safety signals, and mechanistic insights across systemic therapies in patients with psoriasis or PsA who also exhibit ANA positivity, CLE, or SLE. A systematic review following PRISMA 2020 guidelines was conducted across PubMed/MEDLINE, Embase, the Cochrane Library, Scopus, and ClinicalTrials.gov from database inception through 31 October 2025. Thirty-three eligible reports (29 unique clinical studies; 1429 patients) were included and organized into six prespecified overlap subgroups. Mechanistic and translational studies—including ustekinumab and deucravacitinib SLE trial data and reports of IL-17 inhibitor-associated CLE—were reviewed separately to provide contextual interpretation. IL-23 inhibitors were consistently associated with a favorable cross-disease safety profile, with no clear signal for CLE worsening, SLE flares, or drug-induced autoimmunity. IL-17 inhibitors maintained strong psoriatic efficacy but were associated with an increased frequency of de novo or exacerbated CLE. TNF-α inhibitors showed the strongest association with ANA seroconversion, anti-dsDNA induction, drug-induced lupus, and lupus flares. Ustekinumab demonstrated a stable safety profile across lupus-spectrum disease despite variable efficacy in formal SLE trials. TYK2 inhibition provided dual modulation of IL-23 and type I interferon pathways and showed emerging utility in psoriasis or PsA coexisting with CLE or SLE. Apremilast, methotrexate, and mycophenolate mofetil remained reliable non-biologic systemic options. Phototherapy was associated with potential risk in ANA-positive or lupus-susceptible populations and therefore requires careful consideration. Interpretation is limited by the predominantly observational nature and heterogeneity of the available evidence. IL-23 inhibition and TYK2 inhibition appear to offer a balanced profile of efficacy and lupus-related safety in psoriatic disease complicated by lupus-spectrum autoimmunity. IL-17 inhibitors and TNF-α inhibitors may be associated with higher risk in CLE- or SLE-prone patients and therefore warrant particular caution. Personalized treatment strategies should integrate the relative dominance of psoriatic versus lupus disease, ANA/ENA profile, CLE subtype, and underlying mechanistic considerations. Prospective, biomarker-driven studies are needed to guide therapy in this increasingly recognized overlap population (PROSPERO registration: CRD420251241279).

## 1. Introduction

### 1.1. Background and Immunologic Divergence

Psoriasis and psoriatic arthritis (PsA) are chronic, immune-mediated inflammatory diseases driven predominantly by dysregulation of the IL-23/Th17 axis, with central contributions from IL-23, IL-17A/F, and TNF-α [1,2,3,4,5]. In contrast, systemic lupus erythematosus (SLE) and cutaneous lupus erythematosus (CLE) are prototypical type I interferon-driven autoimmune conditions characterized by plasmacytoid dendritic cell activation, B-cell hyperactivity, autoantibody production, and immune-complex-mediated tissue damage [6,7,8,9]. Although all are autoimmune diseases, psoriatic and lupus-spectrum conditions occupy opposing poles of key immunologic axes, which helps explain why therapies that are highly effective for one end of the spectrum may destabilize the other [9,10,11,12].

Within the lupus spectrum, important mechanistic differences exist between systemic disease and skin-restricted CLE subtypes. SLE is dominated by circulating immune complexes, complement consumption, and multi-organ involvement, reflecting a systemically amplified IFN-I/B-cell program [6,7,13,14,15,16,17]. By contrast, CLE—particularly discoid lupus erythematosus (DLE)—is often “skin-locked,” with dense interface dermatitis, scarring follicular destruction, and a persistently IFN-high, TNF-low milieu in lesional skin [9,18,19,20]. Subacute CLE (SCLE) tends to show stronger Ro/La autoantibody associations and photosensitivity [9,18,19,20], whereas classic localized DLE is more fibrosing and cicatricial. These distinctions are clinically relevant because they may influence how biologic agents that target the Th17/IL-23 axis, B cells, or upstream cytokines differentially affect cutaneous versus systemic lupus biology [9,17,18,19,20].

Several treatment classes commonly used in psoriasis intersect with these pathways in different ways. TNF-α inhibitors are highly effective for psoriasis and PsA but are consistently associated with ANA seroconversion, anti-dsDNA induction, drug-induced lupus, and lupus flares [10,18,21,22,23,24,25]. IL-17 inhibitors provide strong psoriatic skin and joint clearance; however, accumulating reports link them to new-onset or aggravated CLE—particularly disseminated DLE and SCLE—suggesting that IL-17 blockade may shift immune balance toward enhanced type I interferon activity in susceptible skin [26,27,28,29,30,31]. In contrast, IL-23 inhibitors are associated with effective psoriatic control with relatively limited interaction with IFN-I-driven pathways, and no consistent signal for CLE worsening or SLE flares has been reported to date [12,13,14,32].

Other systemic agents occupy more neutral positions along this axis. Ustekinumab, an IL-12/23 inhibitor, has been formally evaluated in Phase II and Phase III SLE trials, demonstrating inconsistent efficacy but a reassuring safety profile without evidence of increased lupus activity [33]. However, dedicated data for CLE—particularly DLE—remain limited, and current evidence supports considering ustekinumab as immunologically neutral in lupus rather than as an active lupus therapy [33,34]. TYK2 inhibition with deucravacitinib is mechanistically attractive because TYK2 lies upstream of both IL-23 and type I interferon signaling; early trials and translational studies suggest improvement in CLE molecular signatures and SLE activity [35,36,37], and isolated real-world reports describe concurrent control of psoriasis, PsA, and SLE [35]. Non-biologic options such as methotrexate (MTX), mycophenolate mofetil (MMF), and apremilast remain important therapeutic anchors: MTX and MMF are foundational in SLE and CLE management [6,11,20], whereas apremilast provides systemic psoriatic control without a clear signal for lupus induction [16].

Phototherapy is traditionally regarded as an effective modality for psoriasis. However, ultraviolet radiation—particularly UVB—can amplify interferon gene signatures, promote keratinocyte apoptosis, and precipitate CLE lesions in genetically predisposed individuals [9,20]. In ANA-high or ENA-positive patients, especially those with established CLE or SLE, this IFN-skewing effect may increase risk and highlights the importance of considering ANA/ENA profiles when planning light-based therapy [20,38].

Finally, some agents commonly used in systemic autoimmunity may adversely affect psoriatic disease. Hydroxychloroquine improves CLE and SLE but has been associated with exacerbation of psoriasis [38,39], and rituximab, a CD20-depleting monoclonal antibody widely used in rheumatology, has been repeatedly reported to induce de novo psoriasis or worsen pre-existing psoriasis [40]. These “reverse” signals further illustrate that therapies optimized for lupus are not automatically compatible with psoriatic biology.

### 1.2. Clinical Dilemma and Objective of This Review

Patients who present with psoriasis or PsA in combination with ANA positivity, CLE, or SLE therefore represent a highly heterogeneous and therapeutically complex subgroup [22,23,24,25,33,34,38,39,41,42,43,44,45]. Agents central to psoriasis or PsA management—such as TNF inhibitors and IL-17 inhibitors—may be associated with photosensitive rashes, de novo CLE, drug-induced lupus, or SLE exacerbations in susceptible individuals [10,18,26,27,28,29,30,31,33,34,38,42,43,44]. Conversely, lupus-directed therapies, including hydroxychloroquine and rituximab, can aggravate psoriatic inflammation or precipitate new-onset psoriasis [38,39,40]. Additional complexity arises from the variable clinical expression of lupus (skin-limited SCLE/DLE versus multi-organ SLE), the spectrum of ANA/ENA serologies, and the expanding use of newer agents such as IL-23 and TYK2 inhibitors in routine psoriasis care [12,13,14,35,36,37].

Despite these challenges, there are currently no dedicated, evidence-based guidelines for systemic treatment of psoriatic disease in the setting of ANA positivity or coexisting lupus-spectrum disease [6,7,8,9,17,20]. Clinicians must therefore integrate heterogeneous data from small cohorts, case series, and mechanistic studies when selecting systemic therapy [22,23,24,25,33,34,38,39,41,42,43,44,45].

The objective of this systematic review is to comprehensively synthesize clinical and mechanistic evidence regarding systemic therapies in adults with psoriasis or PsA who concurrently exhibit isolated ANA positivity, cutaneous lupus erythematosus, or systemic lupus erythematosus. By organizing available data across six prespecified overlap subgroups and incorporating contextual mechanistic insights—including TYK2 inhibition [35,36,37], ustekinumab SLE trials [33], and IL-17 inhibitor-associated CLE [26,27,28,29,30,31]—we aim to propose a pragmatic, phenotype- and pathway-guided framework for systemic treatment selection in this complex overlap population.

## 2. Materials and Methods

### 2.1. Study Design and Reporting Standards

**Figure 1:**
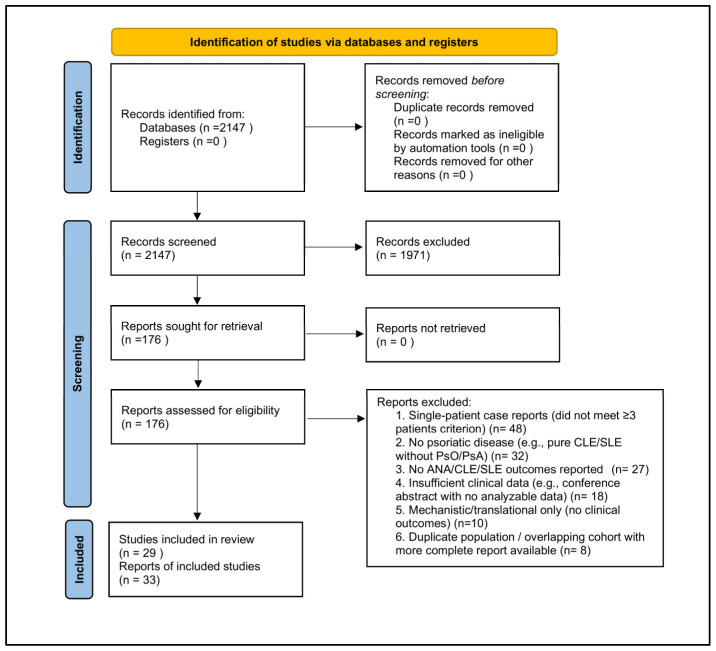
PRISMA 2020 flow diagram illustrating the study identification, screening, eligibility assessment, and inclusion process for this systematic review. A total of 2147 records were identified through database searches. Following title and abstract screening, 1971 records were excluded. The full texts of 176 articles were assessed for eligibility, of which 143 were excluded based on predefined inclusion criteria. Ultimately, 33 studies were included in the qualitative synthesis.

This study is a systematic review conducted in accordance with the PRISMA 2020 statement, following recommended standards for study identification, selection, and transparent reporting. A PRISMA 2020 flow diagram summarizing the study selection process is provided in Figure 1, consistent with PRISMA reporting guidance (Appendix A and Appendix B). The review protocol was prospectively registered in PROSPERO (CRD420251241279).

The objective of this review was to evaluate the reported safety patterns and clinical outcomes of systemic therapies in adults with psoriasis or psoriatic arthritis who also exhibit antinuclear antibody (ANA) positivity, cutaneous lupus erythematosus (CLE), or systemic lupus erythematosus (SLE) [6,7,8,9,17,20]. Given the substantial heterogeneity across available studies—including variability in study design, lupus phenotype definitions, serologic reporting (ANA, ENA, and anti-dsDNA), and outcome measures—a quantitative meta-analysis was not considered appropriate. Accordingly, findings were synthesized using a structured narrative evidence synthesis, consistent with PRISMA guidance for reviews of heterogeneous and predominantly observational data [22,23,24,25,33,34,38,39,41,42,43,44,45].

### 2.2. Eligibility Criteria

#### 2.2.1. Study Eligibility Criteria

Studies were eligible for inclusion if they met all of the following criteria:

Population.

Adults (≥18 years) with psoriasis or psoriatic arthritis and coexisting antinuclear antibody (ANA) positivity, cutaneous lupus erythematosus (CLE), or systemic lupus erythematosus (SLE) [22,23,24,25,32,33,34,38,39,40,41,42,43,44,45,46,47,48,49,50,51,52,53,54,55,56,57,58,59,60,61].

Interventions.

Any systemic therapy used for psoriasis, psoriatic arthritis, or lupus-spectrum disease, including

- Biologic agents targeting TNF-α, IL-17, IL-23, or IL-12/23 pathways [10,18,22,23,24,25,26,27,28,29,30,31,32,46,47,48,49];
- Targeted synthetic therapies such as PDE-4 inhibitors and TYK2 inhibitors [16,35,36,37];
- Conventional immunosuppressive agents, including methotrexate and mycophenolate mofetil [6,11];
- Antimalarial therapies [38,39]; and
- Phototherapy, included for safety and mechanistic context [9,20].

Outcomes.

Psoriasis- and psoriatic arthritis-specific outcomes (e.g., PASI scores, and ACR response criteria), lupus-related outcomes (CLE subtype, CLASI scores, and SLEDAI), serologic trends (ANA titers, and anti-dsDNA changes), lupus flares, CLE induction or worsening, drug-induced lupus, and clinically meaningful composite safety or efficacy endpoints [22,23,24,25,32,33,34,38,39,40,41,42,43,44,45,46,47,48,49,50,51,52,53,54,55,56,57,58,59,60,61].

Study types.

Randomized controlled trials, prospective or retrospective cohort studies, registry analyses, and multi-patient case series (≥3 patients). Case series were included when they reported at least three patients, consistent with methodological frameworks commonly used in the autoimmune dermatology literature [33,34,38,41,42,43].

Exclusion criteria.

Single-patient case reports, pediatric-only studies, in vitro or animal studies, and articles lacking reportable psoriatic or lupus-specific clinical outcomes were excluded [22,23,24,25,33,34,38,42,43].

#### 2.2.2. Standardization of ANA Positivity: Definitions and Clinical Interpretation

##### Definition of ANA Positivity

Given the substantial heterogeneity in how antinuclear antibody (ANA) positivity is defined and reported across the psoriasis–lupus overlap literature, we did not impose a single uniform laboratory threshold for study inclusion. Instead, ANA positivity was accepted as defined by the original studies, reflecting real-world clinical practice and historical variability in laboratory standards [22,23,24,25,51,52,53,54].

Across the included studies, ANA positivity was most commonly determined using indirect immunofluorescence (IIF) on HEp-2 cells, with reported thresholds ranging from ≥1:80 to ≥1:160, and occasionally higher (≥1:320 or ≥1:640) in lupus-enriched cohorts [22,23,24,25,51,52,53,54]. When multiple thresholds were reported within a study, the lowest titer considered clinically positive by the original authors was accepted.

##### Analytical Framework Applied in This Review

To enhance interpretability despite heterogeneous serologic reporting, we applied a conceptual ANA/lupus risk-stratification framework throughout data synthesis and discussion [6,7,8,9,17,20]:

- Isolated ANA positivity:
  Low-to-moderate ANA titers (typically 1:80–1:160) in the absence of clinical lupus manifestations or extractable nuclear antigen (ENA) positivity. This pattern was treated as background autoimmunity, which is relatively common in psoriasis and psoriatic arthritis populations and does not, by itself, imply lupus-spectrum disease [22,23,24,25,51,52,53,54].
- High-risk serologic profile:
  High-titer ANA (≥1:320) and/or positivity for ENA (e.g., anti-Ro/SSA, anti-La/SSB, and anti-dsDNA). This pattern was interpreted as conferring a higher likelihood of interferon-driven lupus-spectrum biology and was analyzed separately when considering therapeutic risk [6,7,8,9,17,20].
- Established lupus-spectrum disease:
  Patients meeting clinical criteria for CLE or SLE, regardless of ANA titer. In these cases, ANA status was considered supportive rather than determinative of diagnosis or risk [6,7,8,9,17,20].

**Figure 2:**
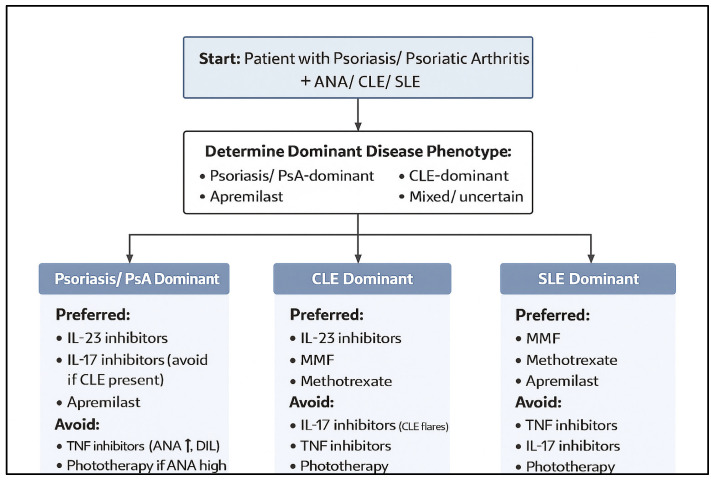
Clinical decision framework for systemic treatment selection in psoriasis and psoriatic arthritis with antinuclear antibody (ANA) positivity, cutaneous lupus erythematosus (CLE), or systemic lupus erythematosus (SLE). The framework begins by identifying the dominant clinical phenotype—psoriasis/PsA-predominant, CLE-predominant, SLE-predominant, or mixed disease—and summarizes systemic therapies according to their relative safety profiles and reported risk patterns in each context. IL-23-targeted therapies are shown as being generally associated with favorable cross-disease safety signals, whereas IL-17 and TNF-α inhibitors are more frequently associated with lupus-related or cutaneous safety concerns in lupus-prone phenotypes. TYK2 inhibition and mycophenolate mofetil are included as options commonly used in SLE-predominant presentations, while phototherapy is depicted as potentially higher risk in CLE/SLE or high-titer ANA states based on photosensitivity and interferon-related considerations.

This stratification framework is explicitly incorporated into the clinical decision framework (Figure 2) and the extended ANA/lupus risk-stratification schema (Supplementary Figure S1).

##### Clinical Interpretation and Limitations

ANA positivity represents a biologic continuum rather than a binary variable. Low-titer ANA positivity is frequently observed in psoriatic disease and may reflect nonspecific immune activation or treatment-associated seroconversion—particularly with TNF-α inhibitors—without progression to clinical lupus [10,18,22,23,24,25]. Conversely, high-titer ANA and ENA positivity are more closely associated with interferon-driven autoimmunity and lupus-spectrum disease [6,7,8,9,17,20].

Accordingly, all analyses and therapeutic interpretations in this review integrate ANA status in conjunction with clinical phenotype (psoriasis/PsA versus CLE versus SLE), rather than treating ANA positivity alone as a contraindication or determinant of therapy. This approach aligns with contemporary rheumatologic and dermatologic practice and mitigates over-interpretation of isolated serologic findings [6,7,8,9,17,20].

### 2.3. Information Sources and Search Strategy

A comprehensive literature search was conducted in PubMed/MEDLINE, Embase, the Cochrane Library, Scopus, and ClinicalTrials.gov from database inception through 31 October 2025. Searches combined controlled vocabulary (MeSH and Emtree terms) with free-text keywords related to psoriasis, psoriatic arthritis, lupus, autoantibodies, and systemic therapies (full strategies provided in Supplementary Table S1).

Key search terms included “psoriasis”, “psoriatic arthritis”, “antinuclear antibody (ANA)”, “cutaneous lupus”, “systemic lupus erythematosus”, “biologic therapy”, “IL-17”, “IL-23”, “TNF inhibitor”, “ustekinumab”, “TYK2 inhibitor”, “phototherapy”, and “drug-induced lupus” [1,2,3,4,5,6,7,8,9,10,11,12,13,14,15,16,17,18,19,20,26,27,28,29,30,31,35,36,37,62,63,64,65].

To identify additional relevant studies, the reference lists of included articles and pertinent reviews were hand-searched [4,9,20,38].

### 2.4. Study Selection

Two reviewers independently screened titles and abstracts for eligibility. Full-text review was undertaken for all records meeting inclusion criteria or when eligibility was uncertain. Any discrepancies were resolved through discussion until consensus was achieved, in accordance with PRISMA 2020 guidance.

A PRISMA 2020 flow diagram summarizing the study selection process is provided in Figure 1.

### 2.5. Data Extraction

Data were extracted using a standardized data-collection template. Extracted variables included study characteristics (author, year, country, and design); patient demographics (age, sex, psoriatic phenotype, and lupus subtype); intervention details (drug class, dose, and duration); psoriatic outcomes (e.g., PASI, ACR response criteria, and joint indices); lupus outcomes (CLE subtype, CLASI, and SLEDAI); serologic measures (ANA titers, anti-dsDNA, and complement levels); and adverse events, including CLE induction or worsening, drug-induced lupus, and SLE flares [22,23,24,25,32,33,34,38,39,40,41,42,43,44,45,46,47,48,49,50,51,52,53,54,55,56,57,58,59,60,61].

When available, mechanistic data were also captured, including type I interferon (IFN-I) gene signatures, TNF-associated autoantibody induction, and transcriptional effects of TYK2 inhibition [9,10,18,26,27,28,29,30,31,35,36,37].

All extracted data were independently cross-verified by two reviewers to ensure accuracy and completeness.

### 2.6. Risk of Bias Assessment

Risk of bias was assessed using design-appropriate tools: the Cochrane Risk of Bias 2.0 tool for randomized controlled trials [33], the Newcastle–Ottawa Scale (NOS) for prospective and retrospective cohort studies [22,23,24,25,51,52,53,54], and the Murad methodological quality tool for case series [33,34,38,41,42,43].

Across study designs, assessments considered key domains including selection methods, comparability of study groups, outcome measurement, follow-up completeness, and reporting transparency. Based on these criteria, studies were categorized as having low, moderate, or high methodological limitations, in accordance with the guidance of each assessment instrument.

Formal quantitative assessments of publication bias (e.g., funnel plots) were not performed, as the included evidence consisted predominantly of heterogeneous observational studies and case series, for which such methods are not methodologically appropriate.

Overall, the included literature most commonly exhibited moderate methodological limitations, reflecting heterogeneity in study design, variability in autoimmune outcome definitions, and inconsistent reporting of serologic measures (e.g., ANA, ENA, anti-dsDNA) across studies [22,23,24,25,32,33,34,38,39,40,41,42,43,44,45,46,47,48,49,50,51,52,53,54,55,56,57,58,59,60,61]. These limitations were taken into account in the interpretation of findings and are discussed further in Section 3.6 and Section 3.19.

### 2.7. Data Synthesis

Given the substantial heterogeneity across interventions, study designs, patient populations, and lupus phenotypes, a quantitative meta-analysis was not considered appropriate. Accordingly, findings were synthesized using a structured narrative approach, consistent with PRISMA guidance for reviews of heterogeneous and predominantly observational evidence.

Results were organized into six clinically relevant overlap subgroups to facilitate interpretable synthesis: (1) psoriasis with ANA positivity, (2) psoriasis with cutaneous lupus erythematosus (CLE), (3) psoriasis with systemic lupus erythematosus (SLE), (4) psoriatic arthritis (PsA) with ANA positivity, (5) PsA with CLE, and (6) PsA with SLE [22,23,24,25,32,33,34,38,39,40,41,42,43,44,45,46,47,48,49,50,51,52,53,54,55,56,57,58,59,60,61].

Contextual and mechanistic evidence—including studies of TYK2 inhibition, reports of IL-17 inhibitor-associated CLE, and analyses of type I interferon pathway activity—was incorporated to inform biologic plausibility and interpretation of safety patterns but was analyzed separately and not included in the primary evidence pool or study counts [9,20,26,27,28,29,30,31,35,36,37].

## 3. Results and Discussion

### 3.1. Study Selection

The systematic search, conducted from database inception through 31 October 2025, identified 2147 unique records after duplicate removal, consistent with comprehensive database strategies described in prior psoriasis–lupus overlap reviews [1,4,9]. Title and abstract screening excluded 1971 records that did not meet inclusion criteria. The full texts of 176 articles were reviewed in detail, of which 143 were excluded for reasons including insufficient clinical data, absence of psoriatic disease, lack of reportable ANA/CLE/SLE outcomes, or single-patient case reports not meeting predefined eligibility criteria [22,23,24,25,34,39,41,44,45]. Ultimately, 33 studies satisfied all inclusion criteria and were incorporated into the qualitative synthesis (Table 1) [22,23,24,25,32,33,34,38,39,40,41,42,43,44,45,46,47,48,49,50,51,52,53,54,55,56,57,58,59,60,61].

A PRISMA 2020 flow diagram summarizing the study selection process is shown in Figure 1, in accordance with PRISMA standards.

In addition to the studies included in the primary qualitative synthesis, contextual and mechanistic sources were reviewed separately to inform biologic plausibility and class-specific safety interpretation. These sources are not part of the formal evidence pool and are described in detail in the Contextual and Mechanistic Evidence subsection (Section 3.3).

### 3.2. Characteristics of Included Studies

The 33 included studies, encompassing 1429 patients, were organized into six predefined clinical subgroups reflecting the intersection of psoriatic disease, ANA serology, and lupus manifestations [22,23,24,25,32,33,34,38,39,40,41,42,43,44,45,46,47,48,49,50,51,52,53,54,55,56,57,58,59,60,61]:

- Psoriasis + ANA positivity (no clinical lupus): 380 patients.
- Psoriasis + cutaneous lupus erythematosus (CLE): 312 patients.
- Psoriasis + systemic lupus erythematosus (SLE): 197 patients.
- Psoriatic arthritis (PsA) + ANA positivity: 326 patients.
- PsA + CLE: 114 patients.
- PsA + SLE: 100 patients.

Across all cohorts, the mean patient age ranged from 35 to 54 years, and 68% were female, consistent with the known predominance of lupus-spectrum disease in women [6,7]. Reported ANA titers demonstrated substantial heterogeneity, ranging from 1:80 to ≥1:640, reflecting variation in baseline autoimmunity, laboratory thresholds, and reporting standards across studies [22,23,24,25,32,46,47,48,49,51,52,53,54].

Among CLE cases, both subacute cutaneous lupus erythematosus (SCLE) and discoid lupus erythematosus (DLE) were represented, allowing stratified interpretation of cutaneous phenotypes within the primary clinical literature [33,34,38,41,42,43]. SLE cohorts included both patients with established lupus and individuals with lupus manifestations temporally associated with systemic therapy exposure, including TNF inhibitor-associated lupus and biologic-associated SLE flares [34,39,43,44,45,50].

Although 29 of the 33 entries represented unique clinical studies, several reports contributed data to multiple subgroups. For example, Prieto-Barrios et al. (2017) [34] and Zalla and Muller (1996) [43] included mixed psoriasis–PsA cohorts encompassing both CLE and SLE phenotypes. Similarly, Ali et al. (2025) [44] and Walhelm et al. (2025) [45] reported SLE-related outcomes in patients with concurrent psoriasis or PsA.

A detailed overview of each study’s design, population, systemic therapy exposure, and key clinical and immunologic findings is provided in Table 2, with full citation mapping to all included primary studies [22,23,24,25,32,33,34,38,39,40,41,42,43,44,45,46,47,48,49,50,51,52,53,54,55,56,57,58,59,60,61].

The contextual and mechanistic literature used to support biologic plausibility and class-specific safety interpretation is described separately in the Contextual and Mechanistic Evidence subsection (Section 3.3) and was not included in the formal evidence pool summarized here.

### 3.3. Contextual and Mechanistic Evidence

#### 3.3.1. Purpose and Scope of Contextual Evidence

In addition to the 33 primary clinical studies included in the formal qualitative synthesis, we reviewed a focused body of contextual and mechanistic evidence to aid interpretation of therapeutic safety patterns and biologic plausibility in psoriasis–lupus overlap disease. This material was not treated as efficacy evidence and was not included in the primary evidence pool, but was analyzed separately to provide interpretive context for observed clinical findings.

Contextual evidence was considered for three specific purposes:

(1) To elucidate biologic mechanisms potentially underlying class-specific safety signals;
(2) To assist interpretation in overlap scenarios where direct clinical evidence is limited; and
(3) To mitigate over-interpretation of findings derived from small observational cohorts or case series.

#### 3.3.2. Types of Contextual Evidence Reviewed

The contextual evidence reviewed in this study comprised the following categories:

1. Randomized controlled trials conducted in lupus-spectrum disease involving agents commonly used in psoriasis or psoriatic arthritis, but not specifically enrolling psoriatic populations (e.g., Phase II and III ustekinumab trials in systemic lupus erythematosus; Phase II deucravacitinib trials in SLE).
2. Mechanistic and translational studies, including transcriptomic analyses and pathway-level investigations (e.g., effects of TYK2 inhibition on type I interferon-regulated gene signatures in cutaneous lupus erythematosus).
3. Published case reports and small case series illustrating rare but biologically informative adverse events (e.g., IL-17 inhibitor-associated cutaneous lupus erythematosus), used solely to identify potential safety signals rather than to estimate incidence or comparative effectiveness.
4. The established pathophysiologic and immunologic literature describing divergence between IL-23/Th17-driven psoriatic inflammation and type I interferon-dominant lupus biology, providing a conceptual framework for cross-disease therapeutic interpretation.

#### 3.3.3. Methodological Handling and Limitations

All contextual sources were explicitly segregated from the primary evidence synthesis and are clearly identified as such in the text, tables, and figure legends. These data were not pooled with cohort or registry outcomes, were not used to generate quantitative comparisons, and did not independently determine therapeutic hierarchies.

We acknowledge that contextual evidence—particularly mechanistic studies and case reports—is inherently subject to selection and publication bias. Accordingly, insights derived from these sources are presented as hypothesis-generating and are intended to support biologic plausibility rather than to establish clinical recommendations.

#### 3.3.4. Integration with Primary Findings

When referenced in subsequent Results and Discussion sections, contextual evidence is used to support interpretation of trends observed in the primary clinical literature, including

- The relative serologic and lupus-related safety signals reported with IL-23 inhibition,
- Associations between IL-17 blockade and cutaneous lupus phenotypes, and
- The mechanistic rationale for TYK2 inhibition in overlap disease involving both Th17- and interferon-driven pathways.

This structured separation between primary clinical evidence and contextual mechanistic data enhances methodological transparency, limits interpretive bias, and aligns the review with best practices for narrative systematic reviews addressing rare or heterogeneous overlap conditions.

### 3.4. Biologic Agents and Exposure Patterns

The following sections synthesize outcomes from the primary clinical studies, interpreted in light of the contextual and mechanistic evidence outlined above. Across the six psoriatic–lupus overlap subgroups, tumor necrosis factor (TNF)-α inhibitors—including etanercept, infliximab, and adalimumab—were the most frequently reported systemic agents, reflecting their long-standing regulatory approval and widespread real-world use [22,23,24,25,51,52,53]. More recently introduced biologic therapies were increasingly represented in newer studies, including IL-12/23 inhibition with ustekinumab [33,34,46], IL-17 inhibition with secukinumab or ixekizumab [26,27,28,29,30,31,47,49], and IL-23 inhibition with agents such as guselkumab or risankizumab [12,13,14,32]. Additional biologics appearing in smaller series or case-level reports included certolizumab pegol, brodalumab, and tildrakizumab, consistent with evolving therapeutic patterns in psoriatic disease [4].

Patterns of ANA monitoring and serologic assessment varied substantially across studies. Some cohorts—such as Pink et al. (2010) [22], Pirowska et al. (2015) [23], and Sugiura et al. (2021) [49]—reported systematic baseline and longitudinal ANA measurements. In contrast, other studies, including Oter-López et al. (2017) [25], García-Arpa et al. (2019) [42], and Ali et al. (2025) [44], focused primarily on clinical lupus manifestations without serial immunologic follow-up. Several reports also described biologic switching, most commonly transitions from anti-TNF therapy to IL-17 or IL-23 inhibitors. Such switches were reported more frequently in individuals with CLE or SLE susceptibility and were often temporally associated with stabilization or improvement of lupus-related cutaneous manifestations [33,34,38,41,42,43,44].

Interpretation of class-specific safety patterns was further supported by contextual mechanistic data external to the primary evidence base, including ustekinumab Phase II/III trials conducted in SLE [33], TYK2 inhibitor immune-signature studies [35,36,37], and published case series describing IL-17 inhibitor-associated CLE [26,27,28,29,30,31]. As detailed in Section 3.3, these sources were used to contextualize biologic plausibility and immunologic mechanisms underlying observed safety signals, rather than to independently define comparative efficacy or therapeutic hierarchy.

### 3.5. Treatment Patterns and Clinical Outcomes Across Subgroups

Therapeutic responses and safety outcomes varied substantially across the six psoriatic–lupus overlap subgroups, reflecting differences in underlying immunobiology and drug-class-specific risk profiles. Key findings for each subgroup are summarized below, with detailed mapping to the original studies [22,23,24,25,32,33,34,38,39,40,41,42,43,44,45,46,47,48,49,50,51,52,53,54,55,56,57,58,59,60,61].

#### 3.5.1. Psoriasis with ANA Positivity (No Clinical Lupus)

Nine studies (*n* = 380) evaluated ANA-positive psoriasis patients: Pink 2010 [22]; Pirowska 2015 [23]; Bardazzi 2014 [24]; Oter-López 2017 [25]; Yanaba 2016 [46]; Miki 2019 [47]; Kutlu 2020 [48]; Sugiura 2021 [49]; and Miyazaki 2023 [32]. ANA seroconversion or titer elevation occurred in approximately 15–35% of patients—most frequently in association with anti-TNF therapy [22,23,24,25]—but remained clinically silent in all reports. No study described CLE, SLE, or drug-induced lupus in this subgroup [22,23,24,25,32,46,47,48,49].

IL-17 and IL-23 inhibitors were consistently associated with stable ANA profiles [32,47,49], indicating relative serologic neutrality. These findings are concordant with ANA-positive PsA cohorts treated with TNF inhibitors—Johnson 2005 [52], Silvy 2015 [53], and Viana 2010 [51]—in which ANA increases were observed without progression to CLE or systemic lupus. Importantly, data from biologic-naïve PsA cohorts further demonstrate that baseline ANA positivity is intrinsically common (approximately 30–50%), with 14% exhibiting titers ≥ 1:80 and approximately 3% demonstrating anti-dsDNA antibodies. Collectively, these observations highlight that background autoimmunity is frequent in psoriatic disease and should not be over-interpreted as treatment-induced. Overall, biologic therapy was generally well tolerated, and isolated ANA positivity alone was not associated with subsequent lupus development.

#### 3.5.2. Psoriasis with Cutaneous Lupus (CLE)

Seven studies (*n* = 312) described patients with psoriasis and comorbid SCLE or DLE: Staniszewska 2025 [41]; García-Arpa 2019 [42]; De Souza 2012 [33]; Sachdeva 2020 [38]; Prieto-Barrios 2017 [34]; the CLE subset within Zalla & Muller 1996 [43]; and together with multiple reports of IL-17 inhibitor-associated CLE [26,27,28,29,30,31].

Anti-TNF agents accounted for the majority of CLE flares, typically presenting as de novo TNF-associated CLE or exacerbation of pre-existing lesions [33,34,38,42,43]. Discontinuation of anti-TNF therapy was frequently followed by clinical improvement, consistent with established patterns of TNF inhibitor-associated lupus phenomena [10,18]. In contrast, IL-12/23, IL-17, and IL-23 inhibitors were reported to have neutral or occasionally favorable cutaneous outcomes, with several reports describing partial or complete CLE improvement following transition away from anti-TNF therapy [34,41]. No cases of new-onset systemic lupus were reported in this subgroup [33,34,38,41,42,43].

#### 3.5.3. Psoriasis with Systemic Lupus Erythematosus (SLE)

Six studies (*n* = 197) examined psoriasis patients with established SLE: Prieto-Barrios 2017 [34]; Zalla & Muller 1996 [43]; Hays 1984 [50]; Tselios 2017 [39]; Ali 2025 [44]; and Walhelm 2025 [45].

Anti-TNF exposure was intermittently associated with SLE flares, dsDNA elevation, cytopenias, or CLE lesions [34,39,43,44,45,50], consistent with known drug-induced lupus mechanisms [10,18]. In contrast, IL-12/23 and IL-17 inhibitors were generally associated with stable serologic and clinical SLE indices in reported cases [34,39,44]. Mechanistic and trial-level evidence from ustekinumab and TYK2 inhibitor studies in SLE [33,35,36,37] provides biologic support for the use of non-TNF-targeted therapies in this setting. Taken together, these findings indicate that TNF-α inhibitors may pose increased risk in psoriasis patients with coexisting SLE and therefore warrant particularly careful consideration.

#### 3.5.4. Psoriatic Arthritis with ANA Positivity

Five studies (*n* = 326) evaluated ANA-positive PsA patients treated predominantly with TNF inhibitors: Johnson 2005 [52]; Silvy 2015 [53]; Viana 2010 [51]; Kara 2025 [54]; and Eibl 2023 [55]. Importantly, ANA positivity is frequently observed in PsA independent of therapy. In the biologic-naïve cohort described by Johnson et al. [52], 47% of untreated PsA patients were ANA-positive (≥1:40), with 14% exhibiting titers ≥ 1:80 and approximately 3% demonstrating anti-dsDNA antibodies despite no prior immunosuppressive exposure. Similar findings reported by Silvy et al. [53] further confirm high baseline ANA prevalence in PsA.

Although ANA seroconversion or titer elevation was commonly observed in TNF inhibitor-treated cohorts [51,52,53,54], no study reported progression to CLE, SLE, or drug-induced lupus. This pattern parallels findings in ANA-positive psoriasis cohorts [22,23,24,25,32,46,47,48,49] and underscores that TNF-associated autoantibody formation does not necessarily translate into clinically manifested lupus-spectrum disease. Limited available data suggest that IL-17 and IL-23 inhibitors are also associated with serologic stability in ANA-positive PsA patients [32,47,49]. Overall, ANA positivity alone was not predictive of autoimmune complications.

#### 3.5.5. Psoriatic Arthritis with Cutaneous Lupus (CLE)

Four studies (*n* = 114) addressed PsA patients with coexisting CLE: Staniszewska 2025 [41]; Walz LeBlanc 2020 [56]; Ali 2025 [44]; and García-Arpa 2019 [42]. Anti-TNF therapy was again most frequently associated with CLE development or exacerbation, mirroring patterns observed in psoriasis with CLE [33,34,38,42,43].

Several reports described clinical improvement or resolution of CLE following transition to IL-17, IL-23, or IL-12/23 inhibitors while maintaining PsA disease control [41]. Collectively, these observations suggest that anti-TNF therapy may be less suitable in PsA patients with concomitant CLE. Hydroxychloroquine, while beneficial for CLE and SLE, requires cautious use due to its documented potential to exacerbate psoriasis or PsA [38,39].

#### 3.5.6. Psoriatic Arthritis with Systemic Lupus Erythematosus (SLE)

Five studies (*n* = 100) described PsA patients with concomitant SLE: Avriel 2007 [57]; Bonilla 2016 [58]; Korkus 2021 [59]; Sato 2020 [60]; and Venetsanopoulou 2025 [61].

Anti-TNF agents were occasionally associated with SLE flares, whereas IL-12/23 and IL-17 inhibitors were generally reported in association with stable lupus activity [39,60], consistent with mechanistic expectations for agents with limited direct activation of interferon pathways [4,33]. Registry-based analyses further suggest a comparatively favorable safety profile for non-TNF biologics in this overlap population [45,59]. Emerging data indicate that TYK2 inhibition may offer additional therapeutic potential due to its dual modulation of IL-23 and type I interferon signaling [35,36,37].

### 3.6. Comparative Safety Signals

Across the six clinical subgroups, distinct safety patterns were observed among biologic and targeted immunomodulatory therapies.

TNF-α inhibitors demonstrated the most consistent lupus-related safety signal, in keeping with the extensive literature describing TNF inhibitor-associated autoantibody formation and drug-induced lupus [10,18,22,23,24,25,33,34,38,42,43,44,45]. Across included studies, TNF-α inhibitors were associated with the highest reported frequencies of drug-induced lupus, ranging from approximately 6% to 15%, and were frequently linked to dsDNA seroconversion, photosensitive rashes, arthralgia, and hypocomplementemia [10,18,22,23,24,25,33,34,38,42,43]. Several reports described systemic lupus flares in psoriasis and psoriatic arthritis patients receiving anti-TNF therapy, with clinical improvement commonly observed following drug discontinuation [34,39,43,44,45,50]. Collectively, these findings indicate that TNF-α inhibition may be associated with increased lupus-related risk in susceptible populations and therefore warrants careful consideration in such settings.

In contrast, IL-17 inhibitors exhibited a distinct safety pattern. While these agents maintained strong efficacy for psoriasis and psoriatic arthritis [2,4,47,49], they were most frequently associated with cutaneous lupus manifestations, including worsening SCLE or DLE and occasional reports of new-onset CLE [26,27,28,29,30,31]. Mechanistically, IL-17 blockade has been proposed to unmask or amplify type I interferon-driven pathways [9,20], providing a biologically plausible explanation for the predominance of cutaneous lupus phenotypes despite generally stable systemic serologic profiles. These observations suggest that IL-17 inhibition may require heightened vigilance when considered in patients with established CLE or those with serologic or clinical features suggestive of cutaneous autoimmunity.

IL-23 inhibitors were associated with a comparatively favorable safety profile across ANA-positive, CLE, and SLE subgroups. No included study identified drug-induced lupus, CLE exacerbation, or SLE flares clearly attributable to IL-23 blockade [12,13,14,32]. Although evidence in established SLE remains limited, available case-level data and cross-disease mechanistic considerations suggest a relatively stable immunologic profile, consistent with the limited direct interaction between IL-23 signaling and interferon-mediated autoimmunity [12,13,14].

Ustekinumab, which targets both IL-12 and IL-23, showed a similarly reassuring safety pattern. Phase II and Phase III trials conducted in SLE reported no lupus-inducing safety signals, despite variable clinical efficacy [33]. In psoriasis and psoriatic arthritis cohorts, ustekinumab was not associated with CLE or SLE flares and appeared to maintain stability in ANA-positive populations [33,34,46].

The TYK2 inhibitor deucravacitinib provided emerging evidence suggestive of potential mechanistic advantage in lupus-prone populations. In Phase II SLE trials, deucravacitinib was associated with improvement in patient-reported outcomes and attenuation of cutaneous lupus molecular signatures, including reductions in type I interferon-regulated transcripts [35,36,37]. Given that TYK2 signaling lies upstream of both IL-23 and type I interferon pathways, its dual modulation may offer a balanced immunologic effect in patients at risk for autoantibody-mediated disease.

Apremilast demonstrated a consistently benign safety profile across reported overlap scenarios. No study described lupus induction, CLE exacerbation, or SLE flares associated with apremilast therapy [16]. Its mechanism of action, centered on PDE-4 inhibition and downstream cyclic AMP modulation, appears to avoid pathways commonly implicated in autoantibody generation or interferon activation.

Finally, phototherapy exhibited a distinct risk profile. Although widely used in psoriasis [14,63], ultraviolet-based therapies are well recognized to provoke photosensitivity and CLE flares in lupus-prone individuals through UV-mediated keratinocyte apoptosis and neoantigen exposure [9,20]. Even in ANA-positive patients without overt lupus, phototherapy may be associated with increased risk, particularly in the presence of high-titer ANA or ENA positivity (e.g., anti-Ro/La) [20,38]. In patients with CLE or established SLE, alternative systemic or biologic treatment strategies may therefore be more appropriate when feasible (Table 3).

### 3.7. Summary of Therapeutic Suitability by Subgroup

Therapeutic suitability varied substantially across the six psoriatic–lupus overlap subgroups, reflecting distinct immunologic drivers and differences in class-specific safety profiles.

In psoriasis with isolated ANA positivity, IL-23 and IL-17 inhibitors were associated with strong psoriatic efficacy and generally stable serologic behavior [12,13,14,32,47,49], while apremilast provided a non-biologic option with a favorable safety profile in available reports [16]. In contrast, TNF-α inhibitors and higher-intensity phototherapy were more frequently associated with autoantibody induction and photosensitive reactions, suggesting that these approaches may warrant additional caution in ANA-positive populations [10,18,22,23,24,25].

In psoriasis with cutaneous lupus erythematosus (CLE), IL-23 inhibitors, apremilast, and methotrexate were generally reported as well tolerated [12,13,14,16,38]. Anti-TNF agents and IL-17 inhibitors were more commonly associated with CLE induction or exacerbation [26,27,28,29,30,31,33,34,38,42,43]. Hydroxychloroquine, despite its established efficacy in CLE and SLE, requires careful risk–benefit assessment in patients with active psoriasis because of its potential to aggravate psoriatic skin disease [38,39].

For psoriasis with systemic lupus erythematosus (SLE), conventional immunomodulators such as methotrexate and mycophenolate mofetil were frequently used in reported cases [6,11,20], with apremilast and ustekinumab demonstrating reassuring safety profiles in overlap settings [16,33,34,46]. TNF-α inhibitors were more often linked to lupus flares and dsDNA seroconversion [10,18,22,23,24,25,34,39,43,44,50], while hydroxychloroquine posed challenges in patients with active psoriasis. Phototherapy was generally considered unsuitable in established SLE because of photosensitivity risk [20,38].

In psoriatic arthritis (PsA) with ANA positivity, IL-17 and IL-23 inhibitors, along with methotrexate, were associated with effective joint control and relative serologic stability in available studies [6,11,32,47,49]. TNF-α inhibitors, although effective for PsA, were more frequently linked to autoantibody induction [51,52,53,54], which may be relevant when ANA positivity is present.

Among patients with PsA and CLE, methotrexate, mycophenolate mofetil, and IL-23 inhibitors were reported to provide disease control across both articular and cutaneous domains [6,11,12,13,14,41]. IL-17 and TNF-α inhibitors were more often associated with CLE worsening or induction [26,27,28,29,30,31,40,42], while hydroxychloroquine again required cautious use because of the risk of psoriatic flaring [38,39].

Finally, in PsA with systemic lupus erythematosus, methotrexate and mycophenolate mofetil commonly served as foundational therapies [6,11], with apremilast offering an additional oral option with a favorable lupus-related safety profile [16]. Emerging evidence suggests that TYK2 inhibition may hold potential in this subgroup because of its dual modulation of IL-23 and type I interferon pathways [35,36,37]. TNF-α inhibitors were more frequently associated with lupus flares in this setting [10,18,45,59], while hydroxychloroquine again required careful consideration when psoriatic disease was active [38,39].

**Figure 3:**
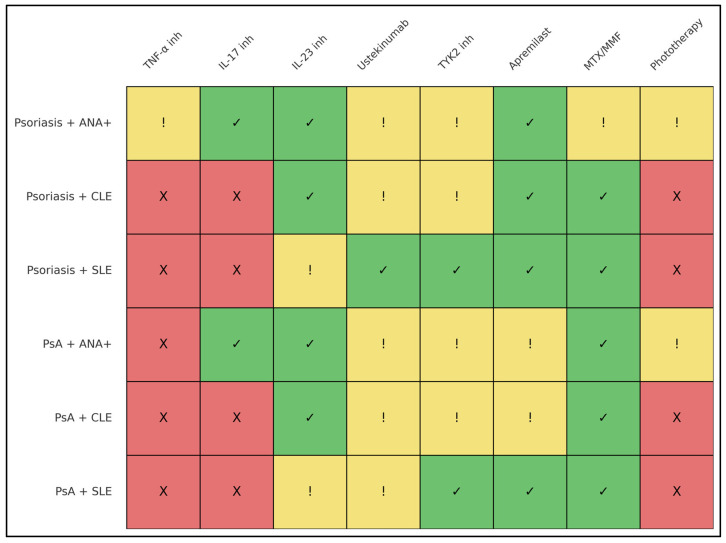
Safety profile of systemic therapies across psoriatic–lupus overlap subgroups. Heatmap summarizing relative safety and tolerability patterns of major therapeutic classes—TNF-α inhibitors, IL-17 inhibitors, IL-23 inhibitors, ustekinumab, TYK2 inhibitors, apremilast, methotrexate/mycophenolate, and phototherapy—across six clinical overlap phenotypes: psoriasis with antinuclear antibody (ANA) positivity, psoriasis with cutaneous lupus erythematosus (CLE), psoriasis with systemic lupus erythematosus (SLE), psoriatic arthritis (PsA) with ANA positivity, PsA with CLE, and PsA with SLE. Color shading reflects reported associations and relative safety considerations in the available literature: green indicates therapies more frequently associated with favorable safety profiles in the corresponding context; yellow indicates therapies used in selected or conditional clinical scenarios; and red indicates therapies more often associated with lupus-related or cutaneous safety concerns. Across overlap phenotypes, IL-23-targeted therapies are shown as being generally associated with favorable cross-phenotype safety signals, whereas TNF-α and IL-17 inhibitors are more frequently associated with lupus-related adverse events in lupus-prone settings. Legend: Green indicates therapies more frequently associated with favorable safety profiles in the corresponding context; yellow indicates therapies used in selected or conditional clinical scenarios; red indicates therapies more often associated with safety concerns or adverse lupus-related outcomes in the available literature.

Taken together, these subgroup-specific patterns suggest a gradual spectrum of relative suitability among systemic therapies in lupus-susceptible psoriatic disease, with IL-23-targeted and interferon-modulating approaches generally associated with more favorable safety signals, and TNF-α inhibition more frequently linked to lupus-related adverse events. Importantly, these observations are derived from heterogeneous and predominantly observational data and should be interpreted as descriptive trends rather than prescriptive treatment algorithms, with further stratification required according to the presence and severity of CLE or SLE (Table 4 and Table 5, and Figure 3).

### 3.8. Discussion

Despite substantial differences in their underlying immunopathogenesis, an increasing body of evidence suggests that patients with psoriatic disease may have an elevated likelihood of developing lupus erythematosus [66,67,68,69]. Epidemiologic studies have consistently reported a higher prevalence of systemic lupus erythematosus (SLE) among individuals with psoriasis or psoriatic arthritis, pointing to potential biological overlap between Th17-driven and type I interferon-driven autoimmune pathways. Experimental models further support this concept: epicutaneous imiquimod application—commonly used to induce psoriasis-like inflammation—has also been shown to provoke lupus-like immune activation in susceptible settings, illustrating how a single upstream trigger can engage both psoriatic and lupus-associated pathways [70,71]. In addition, shared genetic susceptibility loci identified in the Chinese Han population reinforce the notion of a partially overlapping hereditary background linking these two disorders [71].

Within this context, the present systematic review offers a comprehensive synthesis of available evidence regarding the management of psoriasis and psoriatic arthritis in patients who exhibit antinuclear antibody positivity or coexisting cutaneous or systemic lupus erythematosus. By integrating recent mechanistic and clinical findings—including controlled trials of ustekinumab in SLE [33], emerging data on TYK2 inhibition [35,36,37], and accumulating reports of IL-17 inhibitor-associated cutaneous lupus manifestations [26,27,28,29,30,31]—this review seeks to clarify therapeutic patterns and safety considerations in an area historically characterized by clinical complexity and immunologic heterogeneity, rather than to establish prescriptive treatment recommendations.

### 3.9. The Central Immunologic Paradox of Psoriasis–Lupus Overlap

Psoriasis and psoriatic arthritis are driven predominantly by IL-23-mediated activation of Th17 cells and downstream production of IL-17A and IL-17F [1,2,3,4,5], whereas lupus and lupus-spectrum diseases are characterized by plasmacytoid dendritic cell activation and amplification of type I interferon programs that promote B-cell hyperactivity and autoantibody production [6,7,8,9,17]. These conditions therefore occupy opposing ends of key immunologic axes [9,10,11,12]. As a result, therapies developed for psoriasis or psoriatic arthritis may, in some contexts, perturb lupus-associated immune pathways, while treatments effective for lupus may exacerbate psoriatic inflammation [10,18,38,39,40]. This immunologic tension is reflected in the heterogeneous safety profiles observed across biologic classes in the present review.

Antinuclear antibody (ANA) positivity introduces additional complexity. Elevated ANA levels may reflect background autoimmunity, a therapy-associated serologic phenomenon—particularly in the context of TNF-α inhibition [10,18,22,23,24,25]—or, in some cases, an early marker of evolving lupus-spectrum disease [6,7,8,9,17,20]. The clinical significance of ANA positivity therefore varies according to titer, associated autoantibodies, and the presence or absence of clinical lupus manifestations.

Taken together, these considerations suggest that therapeutic decision-making in psoriasis–lupus overlap disease may benefit from an approach that integrates not only diagnostic category but also dominant clinical phenotype, serologic activity, and individualized risk assessment, rather than reliance on disease labels alone.

### 3.10. Differential Implications for SLE Versus CLE, Particularly DLE

**Figure 4:**
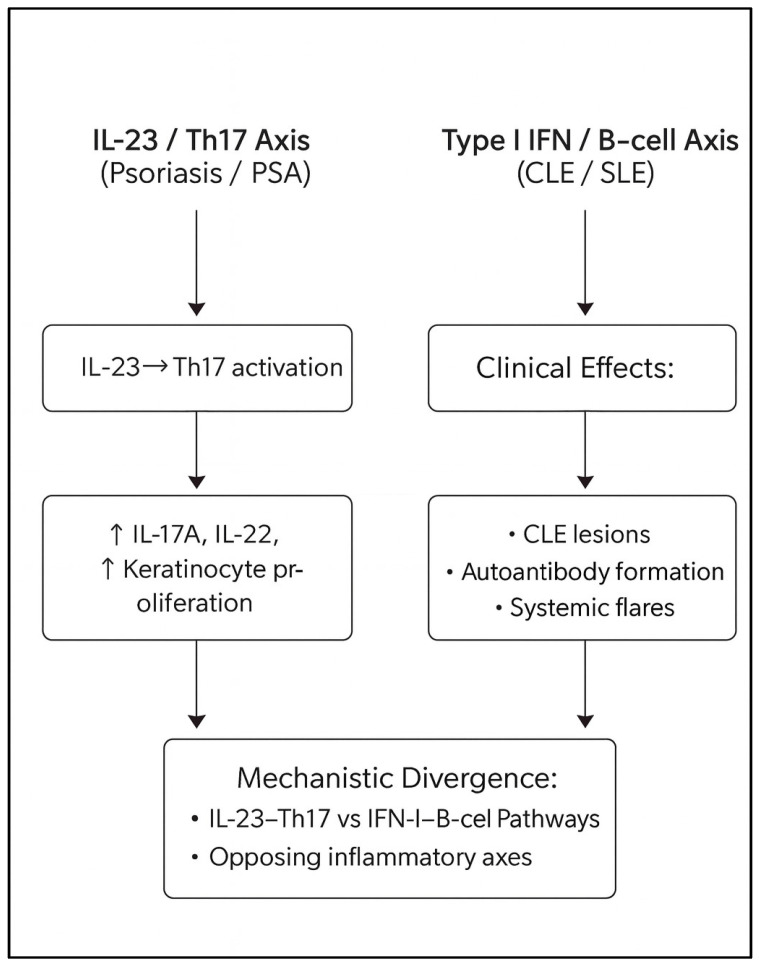
Mechanistic divergence between psoriatic (IL-23/Th17) and lupus (type I interferon/B-cell) immune pathways. Psoriasis and psoriatic arthritis are predominantly associated with IL-23-mediated Th17 activation, with downstream production of IL-17A/F and IL-22 contributing to keratinocyte proliferation and synovial inflammation. In contrast, cutaneous and systemic lupus erythematosus are characterized by plasmacytoid dendritic cell activation and type I interferon signaling, leading to BAFF-mediated B-cell activation, ANA and anti-dsDNA autoantibody production, and CLE/SLE manifestations. These two immune axes illustrate distinct and partially opposing inflammatory programs, which may help explain differential therapeutic responses and observed cross-disease safety patterns across systemic treatments.

Although systemic lupus erythematosus (SLE) and cutaneous lupus erythematosus (CLE) share a type I interferon-driven immunologic core [6,7,8,9,17], their therapeutic vulnerabilities differ in important ways. SLE is characterized by circulating immune complexes, complement activation, and multi-organ inflammation; accordingly, therapies that modulate global B-cell activity (e.g., rituximab) [40] or attenuate type I interferon signatures may provide benefit in selected patients. In contrast, chronic discoid lupus erythematosus (DLE) is frequently “skin-restricted,” with scarring follicular damage, dense interface dermatitis, and a persistently IFN-high, TNF-low cutaneous microenvironment [9,20]. In this context, additional suppression of Th17 or TNF signaling—such as with IL-17 inhibitors [2,4,26,27,28,29,30,31]—has been proposed to further disinhibit plasmacytoid dendritic cell activity and type I interferon production, which may be associated with refractory or exacerbated DLE in susceptible individuals [26,27,28,29,30,31]. This mechanistic framework may help account for observations that IL-17 inhibitor-associated lupus manifestations are more often cutaneous and DLE- or SCLE-like rather than systemic [26,27,28,29,30,31] (Figure 4).

Conversely, agents with neutral or modest interferon-dampening effects, such as ustekinumab [33,46], have demonstrated a stable safety profile in SLE clinical trials despite variable efficacy outcomes [33], and no consistent signal for CLE or DLE worsening has been reported. These findings suggest that ustekinumab may be considered a mechanistically neutral option in psoriatic patients with stable SLE [33,34], while acknowledging the limited availability of CLE-specific data. At the same time, evidence from the broader lupus literature indicates that B-cell-directed therapies such as rituximab, although beneficial for systemic lupus activity, may be associated with paradoxical induction or worsening of psoriasis in some cases [40]. Taken together, these observations underscore the importance of distinguishing SLE from CLE—particularly DLE—when selecting biologic or targeted therapies in patients who span the psoriasis–lupus spectrum [9,17,18,19,20,26,27,28,29,30,31,33].

### 3.11. The Cross-Disease Safety Profile of IL-23 Inhibition

Across the disease combinations examined in this review—including psoriasis with or without CLE or SLE, psoriatic arthritis with or without CLE or SLE, and ANA-positive patients without clinical lupus—IL-23 inhibitors were consistently associated with a favorable balance of reported efficacy and lupus-related safety outcomes [12,13,14,32]. From a mechanistic perspective, the IL-23 blockade acts downstream of pathways implicated in interferon-driven autoimmunity and appears less likely to promote plasmablast activation, anti-dsDNA production, or amplification of type I interferon programs [4,12,13,14].

Within the available clinical literature, no included study reported lupus flares, CLE induction, or drug-induced lupus clearly attributable to IL-23 inhibition [12,13,14,32]. Taken together, these observations suggest that IL-23 inhibitors are generally associated with reassuring safety signals across a broad range of psoriatic–lupus overlap phenotypes, including psoriasis or psoriatic arthritis with ANA positivity [22,23,24,25,32,46,47,48,49,51,52,53,54], psoriasis or psoriatic arthritis with CLE [33,34,38,41,42,43], and stable SLE coexisting with psoriatic disease [34,39,43,44,45,50]. This pattern contrasts with the more frequent lupus-related safety signals reported with IL-17 and TNF-α inhibitors in similar populations [10,18,26,27,28,29,30,31,33,34,38,42,43,44,45] (Table 6 and Table 7).

### 3.12. IL-17 Inhibitors: High Efficacy with Distinct Cutaneous Lupus Considerations

IL-17 inhibitors are associated with robust control of psoriasis and psoriatic arthritis and, in many studies, demonstrate strong skin clearance and joint suppression relative to other biologic classes [2,4,47,49]. However, the available literature reviewed here identifies a recurring pattern of cutaneous lupus erythematosus (CLE) emergence or exacerbation temporally associated with IL-17 blockade [26,27,28,29,30,31]. Published reports include disseminated discoid lupus erythematosus (DLE) initially misdiagnosed as psoriasis [26,27], worsening of DLE during secukinumab therapy [27], secukinumab-associated subacute CLE (SCLE) [28,29], and ixekizumab-associated CLE [31].

From a mechanistic standpoint, IL-17 inhibition has been proposed to unmask or amplify type I interferon-driven immune activity [9,20]. This shift may promote keratinocyte apoptosis and plasmacytoid dendritic cell activation, potentially facilitating CLE lesion development even in the absence of overt systemic lupus activity [9,20,26,27,28,29,30,31]. While these mechanisms remain inferential, they provide a biologically plausible framework for the predominance of cutaneous manifestations observed in association with the IL-17 blockade.

From a clinical perspective, these observations suggest that IL-17 inhibitors may warrant heightened vigilance in patients with active DLE or SCLE, high-titer ANA, or ENA-positive serology [20,38]. In selected situations—such as severe psoriatic disease with quiescent CLE and limited alternative options—IL-17 inhibition has been reported as feasible with careful monitoring [26,27,28,29,30,31]. Overall, the decision to use IL-17 inhibitors in lupus-prone settings should be individualized, balancing psoriatic disease severity against cutaneous lupus risk and considering available therapeutic alternatives.

### 3.13. TNF-α Inhibitors: Lupus-Related Autoimmunity and Safety Considerations

**Figure 5:**
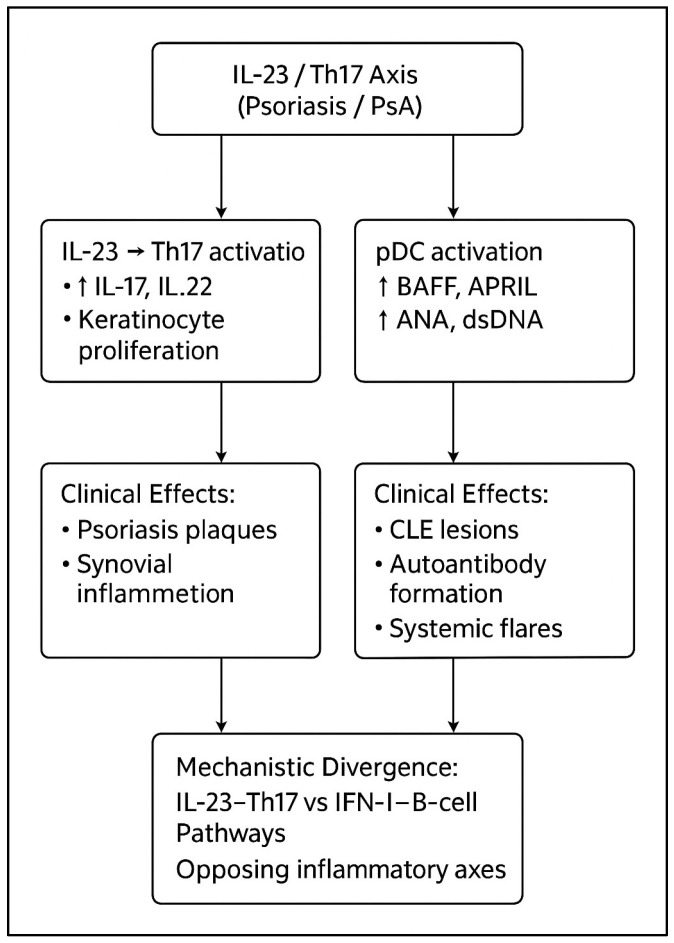
Proposed mechanistic framework for IL-17 inhibitor-associated cutaneous lupus erythematosus (CLE). IL-17 blockade (e.g., secukinumab and ixekizumab) reduces Th17-mediated signaling, which may alter local immune balance in susceptible individuals. This shift has been hypothesized to favor relative type I interferon predominance, potentially involving activation of plasmacytoid dendritic cells and increased IFN-α/β signaling. Downstream effects may include enhanced keratinocyte apoptosis and release of nucleic acid–immune complexes, which have been proposed to amplify autoantibody-associated pathways. Collectively, these processes may contribute to the development of CLE manifestations, such as SCLE or DLE lesions, photosensitive rashes, and occasional ANA or anti-dsDNA elevation, particularly in predisposed patients.

Among the therapeutic classes evaluated, TNF-α inhibitors have been most consistently associated with lupus-related serologic and clinical safety signals in the available literature [10,18,22,23,24,25,33,34,38,42,43,44,45]. ANA seroconversion has been reported in a substantial subset of treated individuals [22,23,24,25,51,52,53,54], anti-dsDNA antibody emergence is well documented [10,18,33,34,38,42,43], and drug-induced lupus has been reported in approximately 6–15% of cases within susceptible populations [10,18,33,34,38,42,43]. In addition, multiple reports describe lupus flares in patients with established SLE receiving anti-TNF therapy [34,39,43,44,45,50], exacerbation of CLE [33,34,38,42,43], and development of new CLE lesions in patients with psoriasis or psoriatic arthritis [33,34,38,42,43]. (Figure 5)

Mechanistic studies provide biologic plausibility for these observations, suggesting that the TNF blockade may promote plasmablast survival, shift immune responses toward interferon-dominant signaling, and facilitate immune complex deposition [10,18]. Taken together, these data indicate that TNF-α inhibition is more frequently associated with lupus-related adverse events in lupus-prone settings, and its use may therefore warrant careful risk–benefit assessment in patients with established CLE/SLE or high-titer ANA positivity [20,38].

### 3.14. Ustekinumab (IL-12/23): Safety Profile in Lupus-Spectrum Disease

Phase II and Phase III clinical trials of ustekinumab in systemic lupus erythematosus provide a relatively robust body of controlled safety data for a biologic agent primarily developed for psoriasis [33]. Although the Phase III trial did not meet its primary efficacy endpoint [33], ustekinumab demonstrated a generally stable safety profile, with no increase in lupus activity or lupus-related adverse events reported during trial follow-up [33].

Within psoriasis and psoriatic arthritis populations, available reports similarly describe stable lupus-related outcomes in ANA-positive patients and in those with coexisting SLE [33,34,46]. These findings suggest that ustekinumab may be considered a mechanistically neutral option in patients whose psoriatic disease is active while lupus manifestations remain stable or mild, particularly in contexts where IL-23-selective agents are unavailable [34]. Its safety profile contrasts with the more frequent lupus-related safety signals reported for TNF-α and IL-17 inhibitors in lupus-susceptible populations [10,18,26,27,28,29,30,31].

### 3.15. TYK2 Inhibition: A Bidirectional Mechanistic Approach

Deucravacitinib, the first approved tyrosine kinase 2 (TYK2) inhibitor, has emerged as a therapy of interest in the context of psoriasis–lupus overlap due to its activity across immune pathways relevant to both disease spectra [35,36,37]. Phase II clinical trial data in systemic lupus erythematosus (SLE) have reported improvements in patient-reported outcomes and attenuation of interferon-driven gene signatures [35,36,37], while experimental and translational studies in cutaneous lupus models have demonstrated suppression of CLE-associated molecular pathways [36]. In addition, early real-world case reports have described clinical improvement in patients with overlapping psoriasis, psoriatic arthritis, and SLE treated with TYK2 inhibition [35].

**Figure 6:**
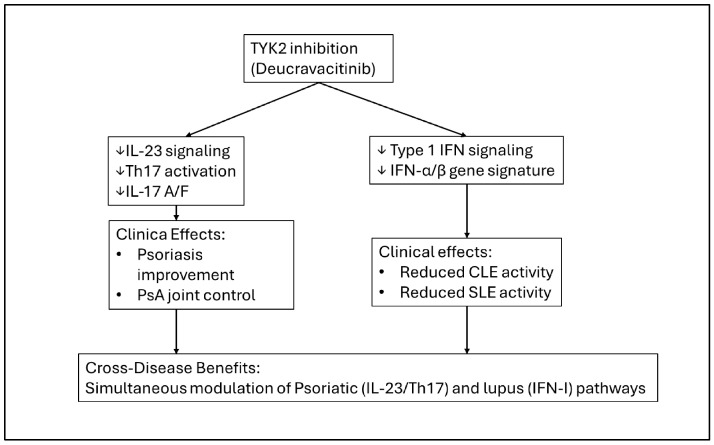
Dual-pathway immunologic effects of TYK2 inhibition in psoriatic–lupus overlap disease. Deucravacitinib inhibits TYK2 signaling upstream of both IL-23-mediated Th17 activation and the type I interferon (IFN-I) axis. Attenuation of IL-23 signaling is associated with reduced Th17 activity and downstream IL-17A/F production, contributing to improvement in psoriasis and psoriatic arthritis manifestations. Concurrent modulation of IFN-α/β-driven pathways is associated with reduced plasmacytoid dendritic cell activation and decreased IFN-I gene signatures, which may correspond with improvement in cutaneous lupus erythematosus (CLE) lesions and stabilization of systemic lupus erythematosus (SLE) activity. Collectively, this dual immunomodulatory effect illustrates a potential mechanistic basis for cross-disease activity of TYK2 inhibition in patients with overlapping psoriatic and lupus-spectrum disease.

From a mechanistic standpoint, TYK2 signaling lies upstream of both IL-23-mediated Th17 activation and type I interferon pathways. Its inhibition therefore provides a theoretically balanced immunomodulatory effect in patients whose disease biology reflects contributions from both Th17-driven and interferon-driven processes [35,36,37]. Taken together, these findings suggest that TYK2 inhibition may represent a promising oral therapeutic option with cross-disease relevance in psoriatic–lupus overlap, although further prospective and real-world studies are needed to better define its role across heterogeneous overlap phenotypes (Figure 6 and Table 8).

### 3.16. Phototherapy: A Reassessment in ANA-Positive and CLE-Prone Disease

Although narrowband UVB phototherapy has traditionally been regarded as an effective and generally well-tolerated modality for psoriasis [14,63], accumulating mechanistic evidence suggests that ultraviolet exposure may amplify type I interferon-regulated gene signatures [9,20], promote keratinocyte apoptosis, and facilitate the development or exacerbation of cutaneous lupus erythematosus (CLE) in genetically or serologically predisposed individuals [20,38]. These biologic observations are concordant with clinical reports describing disease worsening in systemic lupus erythematosus (SLE) and active CLE following phototherapy exposure [20,38].

Patients with high-titer antinuclear antibody (ANA) or extractable nuclear antigen (ENA) positivity may be at increased susceptibility to these effects [20,38], even in the absence of overt clinical lupus manifestations. In contrast, phototherapy has been reported as feasible in selected ANA-low individuals without CLE or SLE features; however, its use in such settings may warrant closer monitoring and individualized risk assessment compared with routine psoriasis populations [9,20]. Taken together, these considerations support a more cautious and phenotype-informed approach to phototherapy in patients with psoriatic disease who exhibit lupus-prone serologic or clinical features (Table 9).

### 3.17. Treatment Strategy by Disease Combination

The findings of this review suggest that therapeutic considerations in psoriatic–lupus overlap disease may benefit from a phenotype-informed, context-dependent approach across the six clinical subgroups.

In psoriasis with isolated ANA positivity, IL-23 inhibitors, IL-17 inhibitors, and apremilast have been reported as generally well tolerated and effective for psoriatic disease control [12,13,14,16,32,47,49]. In contrast, TNF-α inhibitors and phototherapy have been more frequently associated with autoantibody induction or photosensitive reactions, indicating that their use may warrant additional caution in ANA-positive settings [10,18,22,23,24,25].

In psoriasis with cutaneous lupus erythematosus (CLE), IL-23 inhibitors, apremilast, and methotrexate have been described as providing stability across psoriatic and cutaneous disease domains in available reports [6,11,12,13,14,16]. Conversely, IL-17 inhibitors and TNF-α inhibitors have been more commonly associated with CLE induction or exacerbation [26,27,28,29,30,31,33,34,38,42,43].

For psoriasis with systemic lupus erythematosus (SLE), conventional immunomodulators such as mycophenolate mofetil and methotrexate, along with apremilast, ustekinumab, and emerging TYK2 inhibition, have demonstrated reassuring safety profiles in overlap contexts [6,11,16,33,35,36,37,46]. In contrast, TNF-α inhibitors, IL-17 inhibitors, and phototherapy have been more frequently linked to lupus-related adverse events in this population [10,18,20,26,27,28,29,30,31].

In psoriatic arthritis (PsA) with ANA positivity, IL-17 and IL-23 inhibitors and methotrexate have been associated with effective joint control and relative serologic stability in reported studies [2,4,6,11]. TNF-α inhibitors, while effective for PsA, have been more frequently linked to autoantibody induction, which may be relevant in ANA-positive patients [51,52,53,54].

Among patients with PsA and CLE, mycophenolate mofetil, methotrexate, and IL-23 inhibitors have been reported to provide cross-domain disease control across joint and cutaneous manifestations [6,11,12,13,14,41]. In contrast, IL-17 inhibitors, hydroxychloroquine, and TNF-α inhibitors have been more often associated with CLE worsening or psoriatic flares in this subgroup [26,27,28,29,30,31,38,39,40,42].

Finally, in PsA with systemic lupus erythematosus, methotrexate, mycophenolate mofetil, apremilast, and TYK2 inhibition have been described as aligning favorably with both joint and lupus-related disease mechanisms [6,11,16,35,36,37]. TNF-α inhibitors and hydroxychloroquine, by contrast, have been more frequently associated with challenges in the setting of active psoriatic disease [10,18,38,39].

Taken together, these observations underscore the importance of individualized treatment selection, integrating dominant clinical phenotype, serologic profile, and reported safety patterns rather than relying on uniform therapeutic hierarchies.

### 3.18. Therapeutic Scope and Evidence Balance in Psoriasis–Lupus Overlap Disease

#### 3.18.1. Rationale for a Biologic-Focused Synthesis

The present review places substantial emphasis on biologic and targeted synthetic therapies. This focus reflects the contemporary therapeutic landscape of moderate-to-severe psoriasis and psoriatic arthritis, in which biologic agents constitute the predominant escalation strategy and account for a large proportion of reported lupus-related safety signals in the literature [2,4,5,12,13,14]. In addition, biologics targeting the IL-23/Th17 axis or upstream signaling pathways have generated the greatest volume of recent clinical, mechanistic, and pharmacovigilance data relevant to psoriasis–lupus overlap disease [4,12,13,14,35,36,37].

At the same time, we recognize that a biologic-centric emphasis may underrepresent the ongoing clinical importance of non-biologic systemic therapies, particularly in patients with lupus-dominant or mixed phenotypes, where conventional immunomodulators remain integral to disease management [6,11,20].

#### 3.18.2. Role of Non-Biologic Systemic Therapies in Overlap Disease

Conventional systemic agents continue to play essential roles in many psoriasis–lupus overlap scenarios:

- Methotrexate (MTX) remains a foundational therapy for psoriatic arthritis and lupus-associated inflammatory arthritis, offering modest efficacy across skin and joint domains with a long-established safety profile in systemic lupus erythematosus [6,11].
- Mycophenolate mofetil (MMF) represents a cornerstone therapy for both cutaneous and systemic lupus erythematosus and may be particularly valuable in overlap patients with lupus-dominant disease, even when psoriatic manifestations coexist [6,11,20].
- Apremilast, while generally less potent than biologic agents for severe psoriatic skin disease, provides a non-immunogenic oral option with a favorable lupus-related safety profile and may be especially suitable for patients with ANA positivity or mild overlap phenotypes [16].
- Hydroxychloroquine, despite its central role in lupus management, occupies a more nuanced position in overlap disease because of its documented potential to exacerbate psoriasis or psoriatic arthritis. Its use therefore requires careful phenotypic prioritization and close clinical monitoring when psoriatic disease is active [38,39].
- Phototherapy, although effective for psoriasis, warrants particular caution in overlap disease due to its capacity to amplify interferon-mediated pathways and provoke photosensitive lupus manifestations, limiting its applicability in ANA-high or lupus-prone populations [9,20,38].

#### 3.18.3. Integrating Biologic and Non-Biologic Strategies

Rather than positioning biologic and non-biologic therapies as competing categories, the findings of this review support a complementary, phenotype-driven approach. In real-world clinical practice, non-biologic agents frequently function as background or anchoring therapies—either preceding biologic initiation, accompanying targeted agents, or serving as comparatively safer alternatives in patients with heightened lupus risk [6,11,16,20].

Importantly, the relative paucity of lupus-specific safety concerns reported with MTX, MMF, and apremilast underscores their continued relevance, particularly when biologic options are limited by serologic or clinical considerations [6,11,16]. Conversely, the prominence of biologic-associated lupus signals in the literature likely reflects both their widespread use and the degree of immunologic perturbation they may induce in susceptible individuals [10,18,26,27,28,29,30,31].

#### 3.18.4. Implications for Evidence Interpretation

The biologic-weighted evidence base summarized in this review should therefore be interpreted within the context of real-world treatment sequencing, in which conventional systemic therapies often coexist with or precede biologic treatment [6,11,16,20]. The relatively limited representation of non-biologic therapies in comparative analyses reflects gaps in the published literature rather than diminished clinical importance.

Future longitudinal and registry-based studies incorporating both biologic and non-biologic agents will be essential to better define optimal sequencing, combination strategies, and long-term safety outcomes in psoriasis–lupus overlap disease [4,5,20].

### 3.19. The Need for Prospective and Mechanistic Trials

Current knowledge in psoriasis–lupus overlap disease remains constrained by heterogeneous study designs, frequent reliance on case reports and small case series for cutaneous lupus erythematosus (CLE) outcomes [26,27,28,29,30,31,33,34,38,41,42,43], and inconsistent reporting of ANA, ENA, and anti-dsDNA serologies across studies [22,23,24,25,51,52,53,54]. In addition, the absence of large prospective cohorts and randomized controlled trials specifically enrolling patients with psoriatic disease and coexisting lupus-spectrum features limits the ability to draw definitive comparative conclusions regarding therapeutic safety and effectiveness.

Future research efforts would benefit from prioritizing biomarker-informed stratification strategies, including differentiation of IFN-high versus Th17-predominant immunophenotypes [9,17,20]. Prospective evaluation of emerging therapies such as TYK2 inhibitors [35,36,37], targeted studies examining the role of IL-23 blockade in CLE and SLE [12,13,14,32], and mechanistic investigations into IL-17 inhibitor-associated CLE [26,27,28,29,30,31] are particularly needed. Such approaches may help refine patient selection, clarify causal pathways, and improve risk stratification in this complex overlap population.

### 3.20. Clinical Implications

The findings synthesized in this review highlight the importance of a phenotype-informed approach to the management of psoriatic disease coexisting with lupus-spectrum conditions or lupus-prone serology. Across multiple overlap scenarios, IL-23-targeted therapies have been consistently associated with favorable cross-disease safety signals, with no clear evidence of lupus induction in available studies [12,13,14,32]. These observations suggest that IL-23 inhibition may represent a commonly considered biologic option in many overlap contexts, particularly when lupus manifestations are present or serologic risk is a concern.

In contrast, IL-17 inhibitors, while highly effective for psoriatic skin and joint disease, have been more frequently linked to cutaneous lupus emergence or exacerbation, especially in patients with active CLE or photosensitive phenotypes [26,27,28,29,30,31]. Similarly, TNF-α inhibitors remain the therapeutic class most often associated with autoantibody induction and lupus-related adverse events, including ANA and anti-dsDNA seroconversion, drug-induced lupus, CLE flares, and SLE exacerbations [10,18,22,23,24,25,33,34,38,42,43,44,45]. In lupus-prone settings, use of these agents therefore warrants careful risk–benefit assessment and individualized consideration.

Emerging evidence indicates that TYK2 inhibition may offer a mechanistically appealing option in selected patients, given its dual modulation of Th17- and interferon-driven pathways and supportive data from Phase II SLE trials, CLE transcriptomic studies, and early real-world overlap reports [35,36,37]. Phototherapy, while effective for psoriasis in general populations, has been associated with photosensitive lupus flares and interferon-mediated cutaneous activation, underscoring the need for cautious, phenotype-specific use in ANA-positive or lupus-spectrum disease [9,20,38].

Ultimately, therapeutic decision-making in psoriasis–lupus overlap disease is best guided by dominant clinical phenotype (psoriasis/PsA versus CLE or SLE) and the patient’s individual immunologic risk profile, integrating ANA titer, ENA status, and the presence of cutaneous or systemic lupus manifestations [6,7,8,9,17,20]. Rather than relying on uniform treatment hierarchies, these findings support an individualized, mechanism-aware approach that balances disease control with lupus-related safety considerations.

## 4. Conclusions

Patients with psoriasis or psoriatic arthritis who exhibit antinuclear antibody (ANA) positivity or coexistence of cutaneous or systemic lupus erythematosus represent a particularly complex overlap population in clinical immunodermatology. Management in this setting requires careful navigation of two distinct immune programs: the IL-23/Th17 axis that predominates in psoriatic disease [1,2,3,4,5] and the type I interferon–B-cell axis central to lupus pathogenesis [6,7,8,9,17]. The interaction between these immunologic pathways creates context-dependent vulnerabilities when systemic therapies are applied, underscoring the importance of phenotype-informed and mechanism-aware treatment selection [9,10,11,12,20].

Across the clinical and mechanistic evidence synthesized in this review, several consistent patterns can be discerned. IL-23-targeted therapies have been repeatedly associated with a favorable balance of psoriatic efficacy and lupus-related safety across a range of overlap scenarios, with no clear signal for autoantibody induction or lupus activation in the available literature [12,13,14,32]. By contrast, IL-17 inhibitors, while highly effective for psoriatic skin and joint disease, have been more frequently linked to cutaneous lupus manifestations, particularly in patients with SCLE/DLE or interferon-high phenotypes [9,20,26,27,28,29,30,31]. TNF-α inhibitors remain the therapeutic class most often associated with ANA seroconversion, anti-dsDNA induction, anti-TNF-induced lupus, CLE exacerbation, and SLE flares [10,18,22,23,24,25,33,34,38,42,43,44,45], highlighting the need for careful risk–benefit assessment in lupus-prone settings.

Emerging data suggest that TYK2 inhibition, exemplified by deucravacitinib, may offer a mechanistically appealing approach in selected patients by modulating both IL-23-mediated Th17 activity and type I interferon signaling, with supportive evidence from Phase II SLE studies, CLE transcriptomic analyses, and early real-world overlap reports [35,36,37]. In parallel, non-biologic systemic agents—including methotrexate, mycophenolate mofetil, and apremilast—continue to play important roles, particularly in SLE-dominant or mixed phenotypes, owing to their established safety profiles and complementary mechanisms of action [6,11,16]. Phototherapy, although effective for psoriasis in general populations, has been associated with photosensitive lupus activation and interferon-mediated cutaneous risk, indicating that its use in ANA-positive or lupus-spectrum disease should be individualized and phenotype-specific [9,20,38].

Taken together, these findings emphasize the need for individualized, phenotype-driven therapeutic decision-making in psoriasis–lupus overlap disease. Treatment selection is best guided by the dominant clinical presentation (psoriasis/PsA versus CLE or SLE), serologic profile (ANA and ENA status), lupus subtype, and underlying immunologic mechanisms [6,7,8,9,17,20]. Rather than supporting a single uniform treatment hierarchy, the evidence summarized here suggests that IL-23-targeted therapies and, increasingly, TYK2 inhibition are frequently associated with reassuring safety signals and mechanistic alignment in overlap populations, while acknowledging that prospective, biomarker-guided studies are needed to further refine therapeutic strategies in this challenging clinical interface [12,13,14,32,35,36,37].

## Figures and Tables

**Table 1:** Summary of methodological quality across all included studies, assessed using the Cochrane Risk of Bias 2.0 tool for randomized controlled trials, the Newcastle–Ottawa Scale for observational studies, and the Murad methodological quality tool for case series. Across the included literature, studies were most frequently characterized by moderate methodological limitations, reflecting heterogeneity in study design, variability in outcome definitions, and limited lupus-specific outcome reporting.

Study Type	Assessment Tool	Number of Studies	Risk of Bias Category	Common Sources of Bias Identified
Randomized Controlled Trials (RCTs)	Cochrane Risk of Bias 2.0	2	Low to moderate	Limited blinding of outcome assessment; relatively small sample sizes within lupus-specific subgroups
Prospective Cohort Studies	Newcastle–Ottawa Scale (NOS)	9	Low to moderate	Variable follow-up completeness; heterogeneity in ANA and CLE outcome definitions; limited adjustment for potential confounders
Retrospective Cohort Studies	Newcastle–Ottawa Scale (NOS)	10	Moderate	Potential selection bias; incomplete documentation of lupus-related outcomes; variability in biologic exposure duration
Registry Studies	Newcastle–Ottawa Scale (NOS)	2	Low to moderate	Incomplete reporting of lupus activity indices; potential reporting bias inherent to registry-based designs
Case Series (≥3 patients)	Murad methodological quality tool	10	Moderate to high	Absence of comparator groups; selective outcome reporting; variability in serologic assessment and diagnostic criteria for CLE/SLE
Overall Summary	—	33 included papers (29 unique studies)	Predominantly moderate	Heterogeneity in study design; variability in lupus outcome reporting; non-standardized ANA thresholds; small subgroup sizes in CLE/SLE populations

**Table 2:** Characteristics of included studies (33 subgroup entries; 29 unique studies).

Subgroup	Study ID (with Inline Reference)	Country	Design	Biologic(s)	Sample Size	Notes
A. Psoriasis + ANA Positivity (No Lupus)	Pink 2010 [22]	UK	Prospective cohort	Etanercept	*n* = 16	ANA induction observed; no reported CLE or SLE
	Pirowska 2015 [23]	Poland	Prospective cohort	Infliximab, Adalimumab	*n* = 30	~20% ANA seroconversion; no clinical autoimmune manifestations
	Bardazzi 2014 [24]	Italy	Cohort	Anti-TNF	*n* = 48	ANA elevation reported; no lupus-like disease
	Oter-López 2017 [25]	Spain	Retrospective cohort	Anti-TNF	*n* = 21	Changes in ANA titers reported
	Yanaba 2016 [46]	Japan	Prospective	Ustekinumab	*n* = 14	Occasional ANA increase without clinical sequelae
	Miki 2019 [47]	Japan	Prospective	Secukinumab	*n* = 10	No lupus-like clinical events reported
	Kutlu 2020 [48]	Turkey	Case series	Anti-TNF; Ustekinumab	*n* = 9	ANA monitored; no subsequent autoimmune disease reported
	Sugiura 2021 [49]	Japan	Cohort	Ixekizumab	*n* = 17	ANA levels remained stable
	Miyazaki 2023 [32]	Japan	Case series	Guselkumab	*n* = 7	ANA elevation reported without CLE or SLE
B. Psoriasis + Cutaneous Lupus (CLE)	Staniszewska 2025 [41]	Poland	Case series	Various	*n* = 4	PsO with CLE; mixed PsA involvement; SCLE/DLE phenotypes
	García-Arpa 2019 [42]	Spain	Case series	Anti-TNF	*n* = 3	CLE temporally associated with anti-TNF exposure; PsA cases included
	De Souza 2012 [33]	Canada	Case series	Anti-TNF	*n* = 2	CLE occurring in temporal association with anti-TNF therapy
	Sachdeva 2020 [38]	USA	Case report/small series	Anti-TNF	*n* = 1	CLE with photosensitive features during anti-TNF therapy
	Prieto-Barrios 2017 [34]	Spain	21-patient cohort	Etanercept, Adalimumab	CLE subset = 2	CLE-like lesions reported in a subset
	Zalla & Muller 1996 [43]	USA	Retrospective chart review	NA	CLE subset = 2	Early documentation of PsO with CLE; PsA also included
C. Psoriasis + Systemic Lupus Erythematosus (SLE)	Prieto-Barrios 2017 [34]	Spain	Mixed cohort	Etanercept, Adalimumab	SLE subset = 4	PsO with coexisting SLE; overlaps with CLE subgroup
	Zalla & Muller 1996 [43]	USA	Retrospective	NA	*n* = 6	PsO with SLE; overlapping CLE/PsA phenotypes
	Hays 1984 [50]	USA	Case series	NA	*n* = 5	Early reports of PsO with SLE
	Tselios 2017 [39]	Canada	Case series	NA	*n* = 4	PsO preceding SLE diagnosis
	Ali 2025 [44]	USA	Cureus	Anti-TNF	*n* = 3	PsO/PsA with SLE; overlapping CLE features
	Walhelm 2025 [45]	Sweden	Lupus Sci Med	Anti-TNF	*n* = 2	SLE flares temporally associated with anti-TNF exposure
D. Psoriatic Arthritis + ANA Positivity	Johnson 2005 [52]	Canada	Cohort	Anti-TNF	*n* = 28	ANA induction observed without clinical lupus
	Silvy 2015 [53]	France	Cohort	Anti-TNF	*n* = 63	ANA elevation reported; no lupus manifestations
	Viana 2010 [51]	Brazil	Cohort	Anti-TNF	*n* = 17	ANA positivity without clinical lupus
	Kara 2025 [54]	Turkey	Case series	Anti-TNF	*n* = 6	ANA-positive PsA; no lupus-spectrum disease
	Eibl 2023 [55]	Germany	EULAR abstract	Anti-TNF	*n* = 54	ANA serologic profiles reported
E. Psoriatic Arthritis + Cutaneous Lupus (CLE)	Staniszewska 2025 [41]	Poland	Case series	Various	PsA + CLE = 2	Derived from PsO + CLE cohort
	Walz LeBlanc 2020 [56]	USA	Case report	Anti-TNF	*n* = 1	CLE temporally associated with anti-TNF therapy
	Ali 2025 [44]	USA	Cureus	Anti-TNF	*n* = 2	PsA with SLE and CLE features
	García-Arpa 2019 [42]	Spain	Case report	Anti-TNF	*n* = 1	PsA with CLE manifestations
F. Psoriatic Arthritis + Systemic Lupus (SLE)	Avriel 2007 [57]	Israel	Case series	NA	*n* = 2	Early reports of PsA with coexisting SLE
	Bonilla 2016 [58]	USA	Retrospective SLE cohort	Various/mixed	*n* = 20	Increased prevalence of PsA among SLE patients
	Korkus 2021 [59]	Israel	Population case–control	Real-world DMARDs/biologics	*n* = 18	Higher SLE prevalence observed in PsA population
	Sato 2020 [60]	Japan	Case report	Secukinumab (IL-17i)	*n* = 1	PsA improvement with stable SLE activity
	Venetsanopoulou 2025 [61]	Greece	Case-based series + review	Various	*n* = 7	Illustrative treatment challenges involving IL-17 axis

**Table 3:** Summary of class-specific safety signals reported across systemic therapies used in psoriatic–lupus overlap disease, including associations with ANA seroconversion, anti-dsDNA induction, cutaneous lupus erythematosus (CLE) induction or exacerbation, systemic lupus erythematosus (SLE) flares and drug-induced lupus, psoriasis flares reported with hydroxychloroquine, and mechanistic considerations related to Th17- and type I interferon-mediated immune pathways.

Therapeutic Class	ANA Seroconversion	dsDNA Induction	CLE Worsening/Induction	SLE Flares/Drug-Induced Lupus (DIL)	Psoriasis/PsA Flares	Other Relevant Safety Notes (Revised)
TNF-α inhibitors	High (15–30%) [22,23,24,25]	Frequent [10,18,33,34,38,42,43]	CLE (DLE/SCLE) reported in temporal association with TNF-α inhibition [33,34,38,42,43]	Higher reported frequencies (≈6–15% DIL; SLE flares reported) [10,18,34,39,43,44,45,50]	None	Strong associations reported with autoantibody activation; use may warrant particular caution in CLE- or SLE-prone populations [10,18,22,23,24,25,33,34,38,42,43,44,45]
IL-17 inhibitors	Low–moderate [47,49]	Rare [26,27,28,29,30,31]	Multiple reports of DLE/SCLE induction or exacerbation [26,27,28,29,30,31]	Very rare [26,27,28,29,30,31]	None	Mechanistically proposed to unmask IFN-dominant pathways; careful consideration may be warranted in patients with active or CLE-prone disease [9,20,26,27,28,29,30,31]
IL-23 inhibitors	Very low [12,13,14,32]	Very low [12,13,14]	No consistent signal reported to date [12,13,14,32]	No SLE flares or DIL reported [12,13,14]	None	Associated with a comparatively favorable cross-disease safety profile in available studies [12,13,14,32]
IL-12/23 inhibitor (ustekinumab)	Very low [33,46]	Minimal [33]	None reported [33,46]	No lupus-inducing signals reported in Phase II/III SLE trials [33]	None	Demonstrated stable safety despite variable efficacy in SLE trials [33,34]
TYK2 inhibitor (deucravacitinib)	Very low [35,36,37]	None [35,36,37]	Improvements in CLE molecular features reported [36]	Potential benefit suggested in Phase II SLE trials [35,36,37]	None	Dual modulation of IL-23 and IFN-I pathways; emerging evidence suggests potential utility in overlap disease [35,36,37]
PDE-4 inhibitor (apremilast)	None [16]	None [16]	None reported [16]	None reported [16]	None	Generally well tolerated across ANA-positive and lupus-spectrum populations in available reports [16]
Methotrexate (MTX)	None [6,11]	None [6,11]	Neutral [6,11]	Longstanding use in SLE with reported reduction in flare frequency [6,11]	None	Commonly used when lupus activity predominates, particularly for inflammatory arthritis [6,11]
Mycophenolate mofetil (MMF)	None [6,11]	None [6,11]	CLE lesion improvement reported [6,11,20]	Established therapy for SLE with protective effects [6,11]	None	Frequently used in SLE-dominant overlap scenarios [6,11,20]
Hydroxychloroquine (HCQ)	None [38,39]	None [38,39]	Beneficial for CLE [38,39]	Standard therapy in SLE [6,11]	Psoriasis/PsA flares reported [38,39]	Use in active psoriasis or PsA may require careful risk–benefit assessment [38,39]
Rituximab	None reported; may reduce pathogenic autoantibodies [40]	Reduction in anti-dsDNA titers reported [40]	Improvement in refractory CLE/SLE skin disease reported [40]	Effective in severe SLE and systemic autoimmune disease [40]	De novo psoriasis or flares reported [40]	Multiple reports describe psoriasis worsening after rituximab, with improvement following withdrawal; immune balance shifts toward Th17 pathways have been proposed [40]
Phototherapy (UVB/NB-UVB)	ANA increase reported in high-titer patients [20]	None	Photo-induced CLE (DLE/SCLE) reported [9,20]	Photosensitive SLE flares reported [9,20]	None	UV exposure enhances IFN-I signaling; application may warrant caution in ANA-high or ENA-positive patients [9,20,38]

**Table 4:** Therapeutic performance across psoriatic–lupus overlap subgroups. Summary of relative suitability and reported safety patterns of systemic therapies across six clinically defined overlap categories: psoriasis with antinuclear antibody (ANA) positivity, psoriasis with cutaneous lupus erythematosus (CLE), psoriasis with systemic lupus erythematosus (SLE), psoriatic arthritis (PsA) with ANA positivity, PsA with CLE, and PsA with SLE. Therapeutic classifications reflect observed clinical outcomes, lupus-related safety signals, ANA/autoantibody trajectories, and mechanistic considerations related to Th17- and type I interferon-mediated immune pathways, and are intended to summarize descriptive trends rather than prescriptive treatment recommendations.

Patient Subgroup	Therapies Associated with More Favorable Safety Profiles	Therapies Used with Conditional or Context-Dependent Consideration	Therapies More Frequently Associated with Safety Concerns
Psoriasis + ANA positivity	IL-23 inhibitors [12,13,14,32]; IL-17 inhibitors [47,49]; apremilast [16]	Methotrexate [6,11]; narrowband UVB in low-titer ANA without lupus features [20]	TNF-α inhibitors [10,18,22,23,24,25]; high-dose or broad-spectrum phototherapy in high-titer ANA or ENA-positive patients [9,20,38]
Psoriasis + CLE	IL-23 inhibitors [12,13,14,32]; apremilast [16]; methotrexate [6,11]	Ustekinumab (IL-12/23) [33,46]; short-term low-dose systemic corticosteroids [6,11]	IL-17 inhibitors (CLE induction or exacerbation reported) [26,27,28,29,30,31]; TNF-α inhibitors [33,34,38,42,43]; hydroxychloroquine in active psoriasis [38,39]; intensive phototherapy [9,20]
Psoriasis + SLE	Mycophenolate mofetil [6,11]; methotrexate [6,11]; apremilast [16]; TYK2 inhibition (deucravacitinib) [35,36,37]; ustekinumab (reassuring safety, variable efficacy) [33]	IL-23 inhibitors in clinically stable SLE [12,13,14]; hydroxychloroquine when psoriasis is mild and closely monitored [38,39]	TNF-α inhibitors [10,18,34,39,43,44,45,50]; IL-17 inhibitors [26,27,28,29,30,31]; phototherapy in established SLE or active CLE [9,20,38]
Psoriatic arthritis + ANA positivity	IL-17 inhibitors [47,49]; IL-23 inhibitors [12,13,14,32]; methotrexate [6,11]	Apremilast [16]; low-dose systemic corticosteroids as bridging therapy [6,11]	TNF-α inhibitors (frequent autoantibody induction reported) [10,18,51,52,53,54]
Psoriatic arthritis + CLE	Mycophenolate mofetil [6,11]; methotrexate [6,11]; IL-23 inhibitors [12,13,14,32]	Ustekinumab [33,34,46]; apremilast [16]	IL-17 inhibitors (CLE flare risk reported) [26,27,28,29,30,31]; TNF-α inhibitors [33,34,38,42,43]; hydroxychloroquine (psoriasis/PsA flares reported) [38,39]
Psoriatic arthritis + SLE	Mycophenolate mofetil [6,11]; methotrexate [6,11]; apremilast [16]; TYK2 inhibition (deucravacitinib) [35,36,37]	IL-23 inhibitors in stable SLE [12,13,14]; hydroxychloroquine with careful monitoring when psoriatic disease is quiescent [38,39]	TNF-α inhibitors [10,18,44,45,59]; hydroxychloroquine during active psoriasis/PsA [38,39]; phototherapy in SLE [9,20]

**Table 5:** Drug suitability matrix summarizing relative safety and tolerability patterns of systemic therapies in psoriasis and psoriatic arthritis with antinuclear antibody (ANA) positivity, cutaneous lupus erythematosus (CLE), or systemic lupus erythematosus (SLE). Each cell reflects the overall balance of reported efficacy and lupus-related safety considerations for a given drug class within a specific overlap phenotype, integrating psoriatic disease control, CLE/SLE safety signals, ANA/autoantibody dynamics, and mechanistic considerations related to Th17- and type I interferon-mediated immune pathways.

Subgroup	TNF-α Inhibitors	IL-17 Inhibitors	IL-23 Inhibitors	Ustekinumab (IL-12/23)	TYK2 Inhibitor	Apremilast	Methotrexate (MTX)	Mycophenolate (MMF)	Hydroxychloroquine (HCQ)	Phototherapy
Psoriasis + ANA+ (no lupus)	More frequently associated with ANA rise and DIL risk [10,18,22,23,24,25]	Generally associated with favorable safety profiles [47,49]	Generally associated with favorable safety profiles [12,13,14,32]	Used in selected contexts [33,46]	Limited overlap data; mechanistically favorable [35,36,37]	Generally well tolerated [16]	Used in selected contexts [6,11]	Infrequently required when SLE absent [6,11]	Use may require monitoring for psoriatic flares [38,39]	NB-UVB may be considered cautiously; higher-risk in ANA-high/ENA+ states [9,20,38]
Psoriasis + CLE	Frequently associated with CLE induction or exacerbation [10,18,33,34,38,42,43]	Frequently associated with CLE risk [26,27,28,29,30,31]	Generally associated with favorable safety profiles [12,13,14,32]	Neutral safety profile in limited data [33,34,46]	Emerging evidence suggests CLE benefit; limited PsO/PsA data [35,36,37]	Generally well tolerated [16]	Commonly used in overlap settings [6,11]	Commonly used in active CLE/SLE [6,11,20]	Requires caution due to psoriasis flare reports [38,39]	Frequently associated with photosensitive CLE risk [9,20]
Psoriasis + SLE	Frequently associated with lupus flares and DIL [10,18,34,39,43,44,45,50]	Frequently associated with CLE/lupus risk [26,27,28,29,30,31]	Used selectively in clinically stable SLE [12,13,14]	Reassuring safety with variable efficacy [33]	Mechanistically aligned with SLE/CLE biology; emerging evidence [35,36,37]	Generally well tolerated [16]	Commonly used in overlap disease [6,11]	Commonly used in overlap disease [6,11]	May be used selectively when psoriasis is mild and monitored [38,39]	Frequently associated with SLE photosensitivity risk [9,20,38]
PsA + ANA+	Frequently associated with autoantibody induction and DIL risk [10,18,51,52,53,54]	Generally associated with favorable joint and serologic profiles [47,49]	Generally associated with favorable safety profiles [12,13,14,32]	Used in selected contexts [33,46]	Limited data; mechanistically favorable [35,36,37]	Used in selected contexts [16]	Commonly used for joint disease [6,11]	Used in selected contexts [6,11]	Requires caution if psoriatic disease active [38,39]	Use limited to low-titer ANA without lupus features [9,20]
PsA + CLE	Frequently associated with CLE risk [10,18,33,34,38,42,43]	Frequently associated with CLE risk [26,27,28,29,30,31]	Generally associated with favorable safety profiles [12,13,14,32]	Used in selected contexts [33,34,46]	CLE benefit reported; PsA data limited [35,36,37]	Used in selected contexts [16]	Commonly used for joint disease [6,11]	Commonly used in CLE/SLE-dominant overlap [6,11,20]	Frequently associated with psoriatic flares [38,39]	Frequently associated with CLE photosensitivity risk [9,20]
PsA + SLE	Frequently associated with lupus flares [10,18,44,45,59]	Frequently associated with CLE/lupus risk [26,27,28,29,30,31]	Used selectively in clinically stable SLE [12,13,14]	Used in selected contexts [33,34,46]	Dual IL-23/IFN-I modulation; emerging overlap data [35,36,37]	Generally well tolerated [16]	Commonly used for joint disease [6,11]	Commonly used in SLE-dominant disease [6,11]	Requires caution when PsA is active [38,39]	Frequently associated with SLE photosensitivity risk [9,20]

**Table 6:** Comparison of the Th17/IL-23 and type I interferon/B-cell immune axes and the ways in which major systemic therapies interact with these pathways. The table illustrates how differential pathway modulation is associated with distinct clinical effects in psoriasis/psoriatic arthritis versus cutaneous and systemic lupus erythematosus, and summarizes interpretive considerations relevant to overlap disease based on reported mechanistic and clinical observations.

Immunologic Axis/Drug Class	Primary Mediators/Targets	Dominant Disease Context	Effect on Psoriasis/PsA	Effect on CLE/SLE	Interpretive Considerations in Overlap Disease
Th17/IL-23 Axis	IL-23, IL-17A/F, IL-22, TNF-α	Psoriasis, PsA	Central to keratinocyte activation and synovial inflammation [1,2,3,4,5]	Indirect and generally secondary role [9,17]	Modulation of this axis is associated with improvement in psoriatic disease; downstream lupus-related effects appear to depend on accompanying changes in IFN-I activity [9,12,13,14,20]
Type I IFN/B-cell Axis	IFN-α/β, BAFF, ANA, dsDNA, immune complexes	CLE, SLE	May be secondarily activated but not a primary driver [6,7,8,9,17]	Central to cutaneous and systemic lupus activity [6,7,8,9,17]	Therapies that amplify IFN-I signaling or autoantibody production have been associated with increased lupus activity, whereas IFN-suppressive strategies are linked to more favorable lupus outcomes [9,17,20]
TNF-α inhibitors	TNF-α blockade	Psoriasis, PsA, RA	Highly effective for skin and joint disease [1,2,3,4,5]	Associated with ANA increase, dsDNA induction, drug-induced lupus, and CLE flares [10,18,22,23,24,25,33,34,38,42,43,44,45]	TNF-α inhibition is frequently associated with lupus-related serologic and clinical events in susceptible populations, which may warrant careful consideration in CLE/SLE-prone or high-risk ANA-positive patients [10,18,22,23,24,25,33,34,38,42,43,44,45]
IL-17 inhibitors	IL-17A/F blockade	Psoriasis, PsA	Very strong skin and joint efficacy [2,4,47,49]	Associated with de novo or exacerbated SCLE/DLE [26,27,28,29,30,31]	IL-17 blockade has been linked to cutaneous lupus manifestations in CLE-prone settings; use may require heightened vigilance in patients with active or high-risk cutaneous lupus phenotypes [26,27,28,29,30,31]
IL-23 inhibitors	IL-23 p19 blockade (upstream of Th17)	Psoriasis, PsA	Robust psoriatic disease control [12,13,14,32]	Neutral or potentially protective; no consistent lupus signal reported to date [12,13,14,32]	IL-23 inhibition has been associated with favorable cross-disease safety signals in available studies and may represent a relatively stable option across overlap phenotypes [12,13,14,32]
IL-12/23 inhibitor (ustekinumab)	p40 blockade	Psoriasis, PsA; studied in SLE	Effective in psoriasis and PsA [46]	Stable safety profile in SLE trials despite variable efficacy [33]	Ustekinumab is generally considered mechanistically neutral in overlap disease and may be used in selected contexts, particularly when IL-23-selective agents are unavailable [33,34,46]
TYK2 inhibitor (deucravacitinib)	TYK2 signaling (IL-23, IFN-I, IL-12 pathways)	Psoriasis; emerging data in SLE/CLE	Effective in psoriasis and PsA [35,36,37]	Suppression of IFN-I signatures and improvement in CLE/SLE endpoints reported [35,36,37]	Dual modulation of Th17 and IFN-I pathways suggests potential utility in overlap disease; evidence remains emerging [35,36,37]
PDE-4 inhibitor (apremilast)	cAMP-mediated cytokine modulation	Psoriasis, PsA	Moderate efficacy [16]	Neutral to mildly favorable effects reported in lupus [16]	Apremilast is generally well tolerated and may serve as an oral option in ANA-positive or lupus-prone patients [16]
Methotrexate (MTX)	Antimetabolite; T- and B-cell modulation	Psoriasis, PsA, SLE arthritis	Effective for joint and skin manifestations [6,11]	Beneficial for SLE musculoskeletal disease [6,11]	MTX is commonly used in SLE-dominant or mixed overlap phenotypes, particularly when inflammatory arthritis is prominent [6,11]
Mycophenolate mofetil (MMF)	Inhibition of lymphocyte proliferation	SLE, CLE	Modest effects on psoriasis/PsA [6,11]	Strong efficacy in SLE and CLE [6,11,20]	MMF is frequently employed in SLE- or CLE-dominant overlap presentations [6,11,20]
Hydroxychloroquine (HCQ)	TLR7/9 and IFN-I modulation	CLE, SLE	Psoriasis exacerbation reported in some patients [38,39]	Beneficial for CLE and SLE [6,11,38,39]	HCQ may be useful in lupus-dominant disease but requires careful risk–benefit assessment when psoriatic disease is active [38,39]
Phototherapy (NB-UVB/UVB)	UV-induced keratinocyte apoptosis and neo-antigen exposure	Psoriasis	Effective for psoriatic skin disease [63]	CLE flares reported via IFN-I upregulation [9,20]	Phototherapy has been associated with photosensitive lupus activity and may be more suitable in low-risk ANA-positive patients without CLE/SLE features [9,20,38]

**Table 7:** Comparative risk summary for drug-induced autoimmunity across systemic therapies.

Therapeutic Class	Primary Drug-Associated Autoimmune Signal(s)	Strength of Evidence	Typical Clinical Phenotype	Reversibility After Drug Withdrawal	Overall Pattern of Drug-Induced Autoimmunity (Revised)
TNF-α inhibitors	ANA seroconversion; anti-dsDNA induction; anti-TNF-induced lupus (ATIL); CLE-like eruptions [10,18,22,23,24,25,33,34,38,42,43,44,45]	High—multiple cohorts, case series, pharmacovigilance data [10,18,22,23,24,25,33,34,38,42,43]	Photosensitive rash; SCLE/DLE-like lesions; arthritis/serositis; ANA ± dsDNA; occasional systemic lupus features [33,34,38,42,43,44,45]	Typically improves or resolves after withdrawal ± corticosteroids or hydroxychloroquine [33,34,38,42,43]	Frequently associated with lupus-related serologic and clinical events in susceptible populations; careful risk–benefit consideration is often warranted in CLE- or SLE-prone patients [10,18,22,23,24,25,33,34,38,42,43,44,45]
IL-17 inhibitors	New-onset or exacerbated CLE (SCLE/DLE); rare lupus-like events [26,27,28,29,30,31]	Moderate—increasing case reports and series [26,27,28,29,30,31]	Disseminated DLE/SCLE; photo-exacerbated plaques; ANA elevation with minimal systemic involvement [26,27,28,29,30,31]	Improvement commonly reported after drug withdrawal; switching to non-Th17 agents described [26,27,28,29,30,31]	Cutaneous lupus manifestations reported with greater frequency in CLE-prone settings; heightened vigilance may be appropriate in active DLE/SCLE [26,27,28,29,30,31]
IL-23 inhibitors	Occasional ANA changes; no consistent lupus/CLE signal [12,13,14,32]	Low—pooled trial data and real-world reports [12,13,14,32]	Isolated autoantibody changes; lupus events rare and not clearly drug-related [12,13,14]	Withdrawal typically not required	Generally associated with reassuring lupus-related safety signals across reported overlap phenotypes [12,13,14,32]
IL-12/23 inhibitor (ustekinumab)	Rare lupus-like or autoimmune phenomena; neutral findings in SLE trials [33,46]	Low–moderate—Phase II/III SLE and psoriasis data [33,46]	Stable SLE activity; no consistent CLE or systemic flares reported [33]	Withdrawal rarely required [33]	Mechanistically neutral safety profile in available data; commonly considered in psoriasis with stable SLE [33,34,46]
TYK2 inhibitor (deucravacitinib)	Reduction in IFN-driven autoimmunity; improvement in CLE/SLE markers reported [35,36,37]	Emerging—early SLE/CLE trials and transcriptomic studies [35,36,37]	Decreased IFN signatures; improved CLE/SLE activity measures; no de novo lupus reported [35,36,37]	Not typically associated with drug-induced autoimmunity	Emerging evidence suggests a favorable immunologic profile in overlap disease; data remain limited [35,36,37]
PDE-4 inhibitor (apremilast)	Minimal autoimmunity signal; rare nonspecific immune events [16]	Low—extensive psoriasis/PsA use; few lupus reports [16]	Mild, nonspecific immune findings; no consistent ANA/CLE/SLE pattern [16]	Generally reversible; therapy often continued	Generally well tolerated in ANA-positive and lupus-prone populations in reported studies [16]
Methotrexate (MTX)	No drug-induced lupus signature; reduction in SLE activity reported [6,11]	Low—long clinical experience [6,11]	Improvement in joint, skin, and systemic inflammatory manifestations [6,11]	Not applicable (not lupus-inducing)	Widely used with a stable autoimmune safety profile, particularly in SLE-dominant overlap phenotypes [6,11]
Mycophenolate mofetil (MMF)	Treatment of SLE/CLE; no drug-induced lupus reported [6,11,20]	Low—standard SLE/CLE therapy [6,11]	Reduction in CLE lesions and SLE activity [6,11,20]	Not applicable (therapeutic rather than inductive)	Commonly employed in SLE/CLE-dominant overlap disease with protective lupus effects [6,11,20]
Hydroxychloroquine (HCQ)	Psoriasis flares; paradoxical psoriatic autoimmunity reported [38,39]	Moderate—multiple psoriasis flare reports [38,39]	New-onset psoriasis or worsening of existing disease; lupus control generally maintained [38,39]	Psoriasis often improves after withdrawal [38,39]	Low lupus risk but notable psoriasis flare risk; individualized assessment needed when psoriatic disease is active [38,39]
Rituximab	Improvement in SLE; paradoxical psoriasis induction reported [40]	Moderate—case reports and series [40]	De novo psoriasis or flares; concurrent SLE improvement [40]	Psoriasis often improves after withdrawal [40]	Low lupus-induction risk but psoriasis exacerbation reported; relevance depends on psoriatic disease activity [40]
Phototherapy (NB-UVB/UVB)	Photo-induced CLE/DLE/SCLE; IFN-signature amplification [9,20,38]	Moderate—well-documented UV-triggered CLE [9,20]	New or worsening CLE in sun-exposed areas; systemic flares less common [9,20,38]	Improvement reported after cessation and photoprotection [9,20]	Cutaneous lupus flares reported in photosensitive or ANA-high individuals; risk varies by phenotype and exposure [9,20,38]

**Table 8:** Key clinical and mechanistic evidence relevant to ustekinumab and TYK2 inhibition (deucravacitinib) in lupus-spectrum disease, including systemic lupus erythematosus (SLE), cutaneous lupus erythematosus (CLE), and reported real-world psoriatic–lupus overlap. The table summarizes study designs, reported efficacy outcomes, safety observations, and interpretive considerations for the use of these agents in patients with psoriasis or psoriatic arthritis in the context of ANA positivity, CLE, or SLE.

Agent	Study/Population	Design and Sample	Key Efficacy Findings in SLE/CLE	Safety Signals	Interpretive Considerations for Psoriasis/PsA with Lupus-Spectrum Disease
Ustekinumab (IL-12/23 inhibitor)	Phase II SLE trial [33]	Randomized, placebo-controlled; moderate-to-severe SLE	Improved SRI-4 responses compared with placebo; benefits observed in musculoskeletal and mucocutaneous domains [33]	No increase in lupus flares; no drug-induced lupus (DIL); no new safety concerns reported [33]	Demonstrates biologic activity in SLE with a stable safety profile; findings suggest potential suitability in psoriasis/PsA patients with stable or mild SLE, particularly when IL-23-selective agents are unavailable [33,34,46]
	Phase III SLE trial [33]	Randomized, placebo-controlled; multicenter SLE cohort	Did not meet the primary BICLA endpoint; modest improvements observed in selected secondary outcomes (e.g., SRI-6) [33]	Safety profile comparable to placebo; no lupus activation reported [33]	Indicates stable safety with variable SLE efficacy; supports consideration of ustekinumab as a mechanistically neutral option in overlap disease rather than as a primary lupus-directed therapy [33,34]
	Case reports/small CLE series [33,34]	SCLE/DLE patients treated off-label	Variable CLE lesion responses, with improvement reported in some refractory cases [33,34]	No CLE worsening or systemic lupus induction reported [33]	Suggests ustekinumab is unlikely to exacerbate CLE in psoriatic patients, although evidence is limited and non-randomized [33,34]
Deucravacitinib (TYK2 inhibitor)	Phase II SLE trial [35,36,37]	Randomized, placebo-controlled; active SLE with multi-organ involvement	Improvements reported in patient-reported outcomes and global disease activity; reductions in IFN-I-driven gene signatures observed [35,36,37]	No DIL signal; no major lupus flares reported; generally well tolerated [35,36,37]	Provides proof-of-concept that TYK2 targeting may modulate SLE activity while influencing IFN-I pathways, which is mechanistically relevant to overlap disease [35,36,37]
	CLE transcriptomic study [36]	CLE patients; mechanistic/transcriptomic analysis	Decreased IFN-I-regulated transcripts and reduced CLE inflammatory signatures observed [36]	No new safety concerns reported [36]	Indicates potential direct effects on CLE-associated molecular pathways, supporting further exploration in overlap settings [36]
	Real-world PsO + PsA + SLE case [35]	Single-patient refractory overlap treated with deucravacitinib plus MMF and HCQ	Concurrent improvement in psoriasis, PsA, and SLE with steroid-sparing effect reported [35]	No lupus activation or psoriasis flare reported [35]	Illustrates feasibility of TYK2 inhibition in complex overlap disease; interpretation limited by single-case design [35]
	Psoriasis Phase III trials (contextual) [35,36,37]	Phase III randomized controlled trials in psoriasis	High PASI-75/90 response rates and durable efficacy reported [35,36,37]	No CLE or SLE signals reported in psoriasis trials [35,36,37]	Confirms strong psoriatic efficacy with no evident lupus-related safety signals in trial populations; relevance to overlap disease remains to be defined in dedicated studies [35,36,37]

**Table 9:** Overview of phototherapy-related safety considerations across antinuclear antibody (ANA)-positive psoriasis, psoriatic arthritis, cutaneous lupus erythematosus (CLE), and systemic lupus erythematosus (SLE). The table summarizes reported efficacy in psoriatic disease, associations with lupus or CLE activation, and context-dependent considerations according to ANA titer, extractable nuclear antigen (ENA) status, and lupus phenotype, based on available clinical and mechanistic evidence.

Clinical Setting	Phototherapy Modality	Psoriasis/PsA Efficacy	Lupus/Autoimmunity Considerations	Context-Dependent Use Considerations
Psoriasis/PsA, ANA-negative	NB-UVB, BB-UVB	High efficacy for plaque psoriasis; useful for PsA with skin-dominant disease [63]	No lupus-specific safety signals reported	Phototherapy is commonly used in the absence of lupus features or autoimmune history
Psoriasis/PsA, low-titer ANA positivity (no ENA, no lupus symptoms)	NB-UVB preferred; avoidance of high cumulative doses	Effective psoriasis control; may reduce need for systemic therapy [63]	Theoretical IFN-I upregulation and autoantibody evolution reported, but clinical risk appears low in available studies [9,20]	NB-UVB may be considered with exposure limitation and clinical monitoring for CLE-like changes or systemic symptoms [9,20]
Psoriasis/PsA, high-titer ANA or ENA positivity (no overt lupus)	NB-UVB only in selected cases; avoidance of PUVA or high-dose UVB	Psoriatic improvement reported, though alternative systemic options are often available [6,11]	Increased susceptibility to UV-provoked autoimmunity and CLE-like changes reported; IFN-I pathway activation described [9,20,38]	Use may be reserved for situations where systemic therapies are unsuitable, with short treatment courses and close dermatologic and serologic monitoring [9,20,38]
Psoriasis with CLE (SCLE or DLE)	NB-UVB, BB-UVB, PUVA	Psoriasis may improve; CLE lesions are typically photosensitive [9,20,38]	CLE flares, new lesion development, and IFN-I amplification frequently reported [9,20]	Phototherapy has been associated with higher cutaneous lupus risk; systemic or biologic alternatives with IFN-compatible profiles are often considered, along with strict photoprotection [6,11,12,13,14,16]
PsA with CLE	NB-UVB, BB-UVB, PUVA	Limited data; some improvement in psoriatic manifestations reported	Similar risk of CLE exacerbation as psoriasis with CLE [9,20,38]	Phototherapy has been associated with CLE worsening; management often prioritizes systemic agents active across joint and cutaneous disease [6,11,12,13,14]
Psoriasis/PsA with SLE (±CLE)	NB-UVB, BB-UVB, PUVA	Psoriatic skin disease may improve; no benefit for systemic lupus activity [63]	Photosensitive SLE and CLE flares well documented; UV exposure is a recognized trigger [9,20,38]	Phototherapy has been associated with lupus activation in many reports; alternative non-UV systemic strategies and rigorous photoprotection are typically emphasized [9,20,38]
Isolated CLE or SLE without psoriasis/PsA	Any UV-based modality	Not indicated for lupus management [6,11]	High frequency of cutaneous and systemic lupus flares reported with UV exposure [9,20,38]	UV-based therapies are generally not used in lupus-only disease; management relies on systemic immunomodulatory therapies compatible with IFN-mediated biology [6,11,20]

## Data Availability

All data supporting the findings of this study are available within the article and its Supplementary Materials. No new datasets were generated.

## Supplementary Materials

The following supporting information can be downloaded at https://www.mdpi.com/article/10.3390/ijms27021093/s1.

## Abbreviations

The following abbreviations are used in this manuscript:

**Abbreviation**	**Full Term**
ANA	Antinuclear antibody
anti-dsDNA	Anti-double-stranded DNA antibody
ATIL	Anti-TNF-induced lupus
CLE	Cutaneous lupus erythematosus
DIL	Drug-induced lupus
DLE	Discoid lupus erythematosus
ENA	Extractable nuclear antigen
HCQ	Hydroxychloroquine
IFN-I	Type I interferon
IL	Interleukin
IL-17i	IL-17 inhibitor
IL-23i	IL-23 inhibitor
IL-12/23i	IL-12/23 inhibitor (ustekinumab)
MMF	Mycophenolate mofetil
MTX	Methotrexate
NB-UVB	Narrowband ultraviolet B phototherapy
PsA	Psoriatic arthritis
PsO	Psoriasis
PUVA	Psoralen plus ultraviolet A therapy

## Appendix A

**Table A1:** PRISMA 2020 checklist for systematic reviews (all item descriptions reproduced verbatim from the official PRISMA 2020 statement).

Section & Topic	Item #	PRISMA 2020 Checklist Item	Location in Manuscript
Title	1	Identify the report as a systematic review.	Title page
Abstract	2	Provide a structured summary in accordance with PRISMA 2020 for Abstracts.	Abstract
Introduction	3	Rationale: Describe the rationale for the review in the context of existing knowledge.	Section 1
	4	Objectives: Provide an explicit statement of the objective(s) the review addresses.	Section 1 (final paragraph)
Methods	5	Eligibility criteria: Specify inclusion and exclusion criteria and how studies were grouped for synthesis.	Section 2.2
	6	Information sources: Specify all databases, registers, and other sources searched, including dates of coverage.	Section 2.3 (Search period: database inception to 31 October 2025)
	7	Search strategy: Present full search strategies for all databases and registers.	Supplementary Table S1
	8	Selection process: Describe methods used to assess study eligibility, including number of reviewers and any automation tools.	Section 2.1
	9	Data collection process: Describe methods used to collect data from included studies.	Section 2.5
	10a	Data items (outcomes): List and define all outcomes for which data were sought.	Section 2.5
	10b	Data items (other variables): List and define all other variables collected (e.g., participant characteristics, interventions).	Section 2.5
	11	Risk of bias assessment: Describe tools and methods used to assess risk of bias.	Section 2.6
	12	Effect measures: Specify effect measures used for each outcome.	Not applicable (qualitative narrative synthesis only)
	13a	Synthesis methods: Describe criteria for eligibility of studies for each synthesis.	Section 2.7
	13b	Synthesis methods: Describe methods for data preparation.	Section 2.7
	13c	Synthesis methods: Describe methods used to tabulate or visually display results.	Table 1, Table 2, Table 3, Table 4, Table 5, Table 6, Table 7, Table 8 and Table 9; Figure 1, Figure 2, Figure 3, Figure 4, Figure 5 and Figure 6
	13d	Synthesis methods: Describe methods used to synthesize results.	Narrative synthesis
	13e	Exploration of heterogeneity: Describe methods used to explore heterogeneity.	Not applicable (heterogeneity not quantitatively assessed)
	13f	Sensitivity analyses: Describe sensitivity analyses conducted.	Not applicable
	14	Reporting bias assessment: Describe methods used to assess risk of reporting bias.	Section 2.6
Results	15	Study selection: Describe the selection process, including numbers screened and reasons for exclusion; include flow diagram.	Section 3.1; Figure 1
	16a	Study characteristics: Present characteristics of included studies.	Table 1
	16b	Excluded studies: Cite studies that were excluded and explain reasons.	Section 3.1
	17	Risk of bias in studies: Present assessments of risk of bias for each included study.	Section 3.6; Table 1
	18	Results of individual studies: Present results for each study.	Section 3; Table 1, Table 2, Table 3, Table 4, Table 5, Table 6, Table 7 and Table 8
	19	Results of syntheses: Present results of all syntheses conducted.	Section 3.2, Section 3.3, Section 3.4, Section 3.5, Section 3.6, Section 3.7, Section 3.8, Section 3.9, Section 3.10, Section 3.11, Section 3.12, Section 3.13, Section 3.14, Section 3.15, Section 3.16, Section 3.17 and Section 3.18
	20	Reporting biases: Present assessments of risk of reporting bias.	Section 3.6
Discussion	23a	Interpretation: Provide a general interpretation of results in the context of other evidence.	Section 3
	23b	Limitations of evidence: Discuss limitations of the included studies.	Section 3.6 and Section 3.19
	23c	Limitations of review processes: Discuss limitations of the review itself.	Section 3.19
	23d	Implications: Discuss implications for practice and future research.	Section 3.20 and Section 4
Other Information	24	Registration and protocol: Provide registration information.	PROSPERO ID
	25	Support: Describe sources of financial or non-financial support.	Funding section
	26	Competing interests: Declare competing interests.	Conflict of Interest section
	27	Availability of data, code, and materials: Describe availability.	Data Availability section

## Appendix B

**Table A2:** PRISMA 2020 abstract checklist.

Item #	PRISMA for Abstracts Checklist Item (Full Wording)	Location
1	Identify the report as a systematic review.	Abstract
2	Provide an explicit statement of the main objective(s) or question(s).	Abstract
3	Eligibility criteria: Specify inclusion and exclusion criteria.	Abstract
4	Information sources: Specify the databases and dates of searches.	Abstract (search period: database inception through 31 October 2025)
5	Risk of bias: Indicate methods used to assess risk of bias.	Abstract
6	Synthesis of results: Indicate methods used for synthesizing results.	Abstract
7	Included studies: Report the number and type of included studies and participants.	Abstract
8	Results: Present a summary of key findings.	Abstract
9	Limitations: Report limitations of the evidence and/or review.	Abstract
10	Interpretation: Provide a general interpretation and implications of the results.	Abstract
11	Funding: Specify the primary source of funding for the review.	Abstract
12	Registration: Provide registration information (e.g., PROSPERO ID).	Abstract

## References

[1] Christophers E. (2001). Psoriasis–epidemiology and clinical spectrum. Clin. Exp. Dermatol..

[2] Mease P.J., McInnes I.B., Kirkham B., Kavanaugh A., Rahman P., van der Heijde D., Landewé R., Nash P., Pricop L., Yuan J. (2015). Secukinumab Inhibition of Interleukin-17A in Patients with Psoriatic Arthritis. N. Engl. J. Med..

[3] Lebwohl M., Strober B., Menter A., Gordon K., Weglowska J., Puig L., Papp K., Spelman L., Toth D., Kerdel F. (2015). Phase 3 Studies Comparing Brodalumab with Ustekinumab in Psoriasis. N. Engl. J. Med..

[4] Ghoreschi K., Balato A., Enerbäck C., Sabat R. (2021). Therapeutics targeting the IL-23 and IL-17 pathway in psoriasis. Lancet.

[5] Ogdie A., Weiss P. (2015). The Epidemiology of Psoriatic Arthritis. Rheum. Dis. Clin. N. Am..

[6] Wallace D.J., Hahn B.H. (2018). Dubois’ Lupus Erythematosus and Related Syndromes.

[7] Petri M., Orbai A.M., Alarcón G.S., Gordon C., Merrill J.T., Fortin P.R., Bruce I.N., Isenberg D., Wallace D.J., Nived O. (2012). Derivation and validation of the Systemic Lupus International Collaborating Clinics classification criteria for systemic lupus erythematosus. Arthritis Rheum..

[8] Doria A., Zen M., Canova M., Bettio S., Bassi N., Nalotto L., Rampudda M., Ghirardello A., Iaccarino L. (2010). SLE diagnosis and treatment: When early is early. Autoimmun. Rev..

[9] Wenzel J. (2019). Cutaneous lupus erythematosus: New insights into pathogenesis and therapeutic strategies. Nat. Rev. Rheumatol..

[10] Ramos-Casals M., Brito-Zerón P., Soto M.J., Cuadrado M.J., Khamashta M.A. (2008). Autoimmune diseases induced by TNF-targeted therapies. Best. Pract. Res. Clin. Rheumatol..

[11] Benucci M., Gobbi F.L., Del Rosso A., Cesaretti S., Niccoli L., Cantini F. (2003). Disease activity and antinucleosome antibodies in systemic lupus erythematosus. Scand. J. Rheumatol..

[12] Papp K.A., Blauvelt A., Bukhalo M., Gooderham M., Krueger J.G., Lacour J.P., Menter A., Philipp S., Sofen H., Tyring S. (2017). Risankizumab versus Ustekinumab for Moderate-to-Severe Plaque Psoriasis. N. Engl. J. Med..

[13] Blauvelt A., Papp K.A., Griffiths C.E., Randazzo B., Wasfi Y., Shen Y.K., Li S., Kimball A.B. (2017). Efficacy and safety of guselkumab, an anti-interleukin-23 monoclonal antibody, compared with adalimumab for the continuous treatment of patients with moderate to severe psoriasis: Results from the phase III, double-blinded, placebo- and active comparator-controlled VOYAGE 1 trial. J. Am. Acad. Dermatol..

[14] Menter A., Strober B.E., Kaplan D.H., Kivelevitch D., Prater E.F., Stoff B., Armstrong A.W., Connor C., Cordoro K.M., Davis D.M. (2019). Joint AAD-NPF guidelines of care for the management and treatment of psoriasis with biologics. J. Am. Acad. Dermatol..

[15] Wallace D.J., Gudsoorkar V.S., Weisman M.H., Venuturupalli S.R. (2012). New insights into mechanisms of therapeutic effects of antimalarial agents in SLE. Nat. Rev. Rheumatol..

[16] Kavanaugh A., Mease P.J., Gomez-Reino J.J., Adebajo A.O., Wollenhaupt J., Gladman D.D., Lespessailles E., Hall S., Hochfeld M., Hu C. (2014). Treatment of psoriatic arthritis in a phase 3 randomised, placebo-controlled trial with apremilast, an oral phosphodiesterase 4 inhibitor. Ann. Rheum. Dis..

[17] Kaul A., Gordon C., Crow M.K., Touma Z., Urowitz M.B., van Vollenhoven R., Ruiz-Irastorza G., Hughes G. (2016). Systemic lupus erythematosus. Nat. Rev. Dis. Primers.

[18] He Y., Sawalha A.H. (2018). Drug-induced lupus erythematosus: An update on drugs and mechanisms. Curr. Opin. Rheumatol..

[19] Mease P.J., Ory P., Sharp J.T., Ritchlin C.T., Van den Bosch F., Wellborne F., Birbara C., Thomson G.T.D., Perdok R.J., Medich J. (2009). Adalimumab for long-term treatment of psoriatic arthritis: 2-year data from the Adalimumab Effectiveness in Psoriatic Arthritis Trial (ADEPT). Ann. Rheum. Dis..

[20] Teboul A., Arnaud L., Chasset F. (2024). Recent findings about antimalarials in cutaneous lupus erythematosus: What dermatologists should know. J. Dermatol..

[21] Zhao M.H., Cons Molina F., Aroca G., Tektonidou M.G., Mathur A., Tangadpalli R., Sun R., Martin R., Pellet P., Huynh T.N.P. (2025). Secukinumab in active lupus nephritis: Results from phase III, randomised, placebo-controlled study (SELUNE) and open-label extension study. Rheumatology.

[22] Pink A.E., Fonia A., Allen M.H., Smith C.H., Barker J.N. (2010). Antinuclear antibodies associate with loss of response to antitumour necrosis factor-alpha therapy in psoriasis: A retrospective, observational study. Br. J. Dermatol..

[23] Pirowska M.M., Goździalska A., Lipko-Godlewska S., Obtułowicz A., Sułowicz J., Podolec K., Wojas-Pelc A. (2015). Autoimmunogenicity during anti-TNF therapy in patients with psoriasis and psoriatic arthritis. Postep. Dermatol. Alergol..

[24] Bardazzi F., Odorici G., Virdi A., Antonucci V.A., Tengattini V., Patrizi A., Balestri R. (2014). Autoantibodies in psoriatic patients treated with anti-TNF-α therapy. J. Dtsch. Dermatol. Ges..

[25] Oter-López B., Llamas-Velasco M., Sánchez-Pérez J., Dauden E. (2017). Induction of Autoantibodies and Autoimmune Diseases in Patients with Psoriasis Receiving Tumor Necrosis Factor Inhibitors. Actas Dermosifiliogr..

[26] Ye B., Liu D., Tu C., Yan S., Liu Y. (2024). Secukinumab-Aggravated Disseminated Discoid Lupus Erythematosus Misdiagnosed as Psoriasis. Cureus.

[27] Hsieh C.Y., Tsai T.F. (2022). Aggravation of discoid lupus erythematosus in a patient with psoriasis and psoriatic arthritis during treatment of secukinumab: A case report and review of literature. Lupus.

[28] Kaler J., Kaeley G.S. (2021). Secukinumab-Induced Lupus Erythematosus: A Case Report and Literature Review. J. Clin. Rheumatol..

[29] Conforti C., Retrosi C., Giuffrida R., Vezzoni R., Currado D., Navarini L., Farinazzo E., Dell’Aquila C., Nan K., Di Meo N. (2020). Secukinumab-induced subacute cutaneous lupus erythematosus. Dermatol. Ther..

[30] Chatzimichail G., Zillikens D., Thaçi D. (2020). Secukinumab-induced chronic discoid lupus erythematosus. JAAD Case Rep..

[31] Ávila-Ribeiro P., Lopes A.R., Martins-Martinho J., Nogueira E., Antunes J., Romeu J.C., Cruz-Machado A.R., Vieira-Sousa E. (2023). Secukinumab-induced systemic lupus erythematosus in psoriatic arthritis. ARP Rheumatol..

[32] Miyazaki S., Ozaki S., Ichiyama S., Ito M., Hoashi T., Kanda N., Saeki H. (2023). Change in Antinuclear Antibody Titers during Biologic Treatment for Psoriasis. J. Nippon Med. Sch..

[33] De Souza A., Ali-Shaw T., Strober B.E., Franks A.G. (2011). Successful treatment of subacute lupus erythematosus with ustekinumab. Arch. Dermatol..

[34] Prieto-Barrios M., Castellanos-González M., Velasco-Tamariz V., Burillo-Martínez S., Morales-Raya C., Ortiz-Romero P., Rivera-Diaz R. (2017). Two poles of the Th 17-cell-mediated disease spectrum: Analysis of a case series of 21 patients with concomitant lupus erythematosus and psoriasis. J. Eur. Acad. Dermatol. Venereol..

[35] Gewiss C., Steinmetz O.M., Roth L., Augustin M., Ben-Anaya N. (2025). Effective management of psoriasis and psoriatic arthritis in a patient with systemic lupus erythematosus using deucravacitinib, mycophenolate mofetil and hydroxychloroquine. Ski. Health Dis..

[36] Wasserer S., Seiringer P., Kurzen N., Jargosch M., Eigemann J., Aydin G., Raunegger T., Schmidt-Weber C.B., Eyerich S., Biedermann T. (2025). Tyrosine kinase 2 inhibition improves clinical and molecular hallmarks in subtypes of cutaneous lupus. Br. J. Dermatol..

[37] Mosca M., Arnaud L., Askanase A., Hobar C., Becker B., Singhal S., Banerjee S., Pomponi S., Choi J., Strand V. (2025). Deucravacitinib, an oral, selective, allosteric, tyrosine kinase 2 inhibitor, in patients with active SLE: Efficacy on patient-reported outcomes in a phase II randomised trial. Lupus Sci. Med..

[38] Sachdeva M., Mufti A., Maliyar K., Lytvyn Y., Yeung J. (2020). Hydroxychloroquine effects on psoriasis: A systematic review and a cautionary note for COVID-19 treatment. J. Am. Acad. Dermatol..

[39] Tselios K., Yap K.S., Pakchotanon R., Polachek A., Su J., Urowitz M.B., Gladman D.D. (2017). Psoriasis in systemic lupus erythematosus: A single-center experience. Clin. Rheumatol..

[40] Wohl Y., Brenner S. (2004). Cutaneous LE or psoriasis: A tricky differential diagnosis. Lupus.

[41] Staniszewska I., Mądrzak L., Piekarczyk H., Baran M., Kalińska-Bienias A. (2025). A Systematic Review of Case Reports on the Co-occurrence of Cutaneous Lupus Erythematosus and Psoriasis. Dermatol. Rev..

[42] García-Arpa M., Flores-Terry M.A., Ramos-Rodríguez C., Franco-Muñoz M., González-Ruiz L., Ramírez-Huaranga M.A. (2020). Cutaneous lupus erythematosus, morphea profunda and psoriasis: A case report. Reumatol. Clin. (Engl. Ed.).

[43] Zalla M.J., Muller S.A. (1996). The coexistence of psoriasis with lupus erythematosus and other photosensitive disorders. Acta Derm. Venereol. Suppl..

[44] Ali M., Abdullah S., Abdulhameed Y., Elbadawi F. (2025). Systemic Lupus Erythematosus and Psoriasis Overlap: A Case Series. Cureus.

[45] Walhelm T., Parodis I., Enerbäck C., Arkema E., Sjöwall C. (2025). Comorbid psoriasis in systemic lupus erythematosus: A cohort study from a tertiary referral centre and the National Patient Register in Sweden. Lupus Sci. Med..

[46] Yanaba K., Umezawa Y., Honda H., Sato R., Chiba M., Kikuchi S., Asahina A., Nakagawa H. (2016). Antinuclear antibody formation following administration of anti-tumor necrosis factor agents in Japanese patients with psoriasis. J. Dermatol..

[47] Miki M., Endo C., Naka Y., Fukuya Y., Kobayashi S., Kawashima M., Tsunemi Y. (2019). Increase in antinuclear antibody levels through biologic treatment for psoriasis. J. Dermatol..

[48] Kutlu Ö., Çetinkaya P., Şahin T., Ekşioğlu H.M. (2020). The Effect of Biological Agents on Antinuclear Antibody Status in Patients with Psoriasis: A Single-Center Study. Indian Dermatol. Online J..

[49] Sugiura R., Terui H., Shimada-Omori R., Yamazaki E., Tsuchiyama K., Takahashi T., Aiba S., Yamasaki K. (2021). Biologics modulate antinuclear antibodies, immunoglobulin E, and eosinophil counts in psoriasis patients. J. Dermatol..

[50] Hays S.B., Camisa C., Luzar M.J. (1984). The coexistence of systemic lupus erythematosus and psoriasis. J. Am. Acad. Dermatol..

[51] Viana V.S., de Carvalho J.F., de Moraes J.C., Saad C.G., Ribeiro A.C., Gonçalves C., Saad S., de Medeiros Ribeiro A.C., Gonçalves C., Bueno C. (2010). Autoantibodies in patients with psoriatic arthritis on anti-TNFα therapy. Rev. Bras. Reumatol..

[52] Johnson S.R., Schentag C.T., Gladman D.D. (2005). Autoantibodies in biological agent naive patients with psoriatic arthritis. Ann. Rheum. Dis..

[53] Silvy F., Bertin D., Bardin N., Auger I., Guzian M.C., Mattei J.P., Guis S., Roudier J., Balandraud N. (2015). Antinuclear Antibodies in Patients with Psoriatic Arthritis Treated or Not with Biologics. PLoS ONE.

[54] Kara M., Alp G. (2025). Antinuclear Antibody Status and Effect of Biological Therapy in Psoriatic Arthritis Patients: A Single-Center Study. Med. J. İzmir Hosp..

[55] Eibl J., Klotz W., Herold M. (2013). AB0277 Antinuclear antibodies in patients with tnf- inhibitors. Ann. Rheum. Dis..

[56] Walz LeBlanc B.A., Gladman D.D., Urowitz M.B. (1994). Serologically active clinically quiescent systemic lupus erythematosus–predictors of clinical flares. J. Rheumatol..

[57] Avriel A., Zeller L., Flusser D., Abu Shakra M., Halevy S., Sukenik S. (2007). Coexistence of psoriatic arthritis and systemic lupus erythematosus. Isr. Med. Assoc. J..

[58] Bonilla E., Shadakshari A., Perl A. (2016). Association of psoriasis and psoriatic arthritis with systemic lupus erythematosus. Rheumatol. Orthop. Med..

[59] Korkus D., Gazitt T., Cohen A.D., Feldhamer I., Lavi I., Haddad A., Greenberg-Dotan S., Batat E., Zisman D. (2021). Increased Prevalence of Systemic Lupus Erythematosus Comorbidity in Patients with Psoriatic Arthritis: A Population-based Case-control Study. J. Rheumatol..

[60] Sato K., Aizaki Y., Yoshida Y., Mimura T. (2020). Treatment of psoriatic arthritis complicated by systemic lupus erythematosus with the IL-17 blocker secukinumab and an analysis of the serum cytokine profile. Mod. Rheumatol. Case Rep..

[61] Venetsanopoulou A.I., Katsigianni I., Skouvaklidou E., Vounotrypidis P., Voulgari P.V. (2025). Uncommon Coexistence of Systemic Lupus Erythematosus and Psoriatic Arthritis: A Case-Based Review. Curr. Rheumatol. Rev..

[62] Aringer M., Costenbader K., Daikh D., Brinks R., Mosca M., Ramsey-Goldman R., Smolen J.S., Wofsy D., Boumpas D.T., Kamen D.L. (2019). 2019 European League Against Rheumatism/American College of Rheumatology classification criteria for systemic lupus erythematosus. Ann. Rheum. Dis..

[63] Kalb R.E., Fiorentino D.F., Lebwohl M.G., Toole J., Poulin Y., Cohen A.D., Goyal K., Fakharzadeh S., Calabro S., Chevrier M. (2015). Risk of Serious Infection With Biologic and Systemic Treatment of Psoriasis: Results From the Psoriasis Longitudinal Assessment and Registry (PSOLAR). JAMA Dermatol..

[64] Gao Y., Zhou Y., Lin Z., Chen F., Wu H., Peng C., Xie Y. (2024). Prioritizing drug targets in systemic lupus erythematosus from a genetic perspective: A druggable genome-wide Mendelian randomization study. Clin. Rheumatol..

[65] Lorenzo-Vizcaya A., Isenberg D.A. (2023). Clinical trials in systemic lupus erythematosus: The dilemma-Why have phase III trials failed to confirm the promising results of phase II trials?. Ann. Rheum. Dis..

[66] Duan K., Wang J., Chen S., Chen T., Wang J., Wang S., Chen X. (2024). Causal associations between both psoriasis and psoriatic arthritis and multiple autoimmune diseases: A bidirectional two-sample Mendelian randomization study. Front. Immunol..

[67] Fijałkowska A., Wojtania J., Woźniacka A., Robak E. (2024). Psoriasis and Lupus Erythematosus-Similarities and Differences between Two Autoimmune Diseases. J. Clin. Med..

[68] Tsai T.F., Wang T.S., Hung S.T., Tsai P.I., Schenkel B., Zhang M., Tang C.H. (2011). Epidemiology and comorbidities of psoriasis patients in a national database in Taiwan. J. Dermatol. Sci..

[69] van der Fits L., Mourits S., Voerman J.S., Kant M., Boon L., Laman J.D., Cornelissen F., Mus A.-M., Florencia E., Prens E.P. (2009). Imiquimod-induced psoriasis-like skin inflammation in mice is mediated via the IL-23/IL-17 axis. J. Immunol..

[70] Hovd A.-M., Kanapathippillai P., Ursvik A., Skagen T., Figenschau S., Bjørlo I.E., von Hofsten S., Holte C., Al-Saad S., Fenton K. (2025). Imiquimod-induced onset of disease in lupus-prone nzb/w f1 mice. J. Rheumatol..

[71] Sun L.D., Cheng H., Wang Z.X., Zhang A.P., Wang P.G., Xu J.H., Zhu Q.X., Zhou H.-S., Ellinghaus E., Zhang F.R. (2010). Association analyses identify six new psoriasis susceptibility loci in the Chinese population. Nat. Genet..

