# Supplementary material for: Systemic Treatment Strategies for Patients with Psoriasis and Psoriatic Arthritis in the Setting of ANA Positivity or Lupus Spectrum Disease: A Comprehensive Systematic Review
Source: Int J Mol Sci. 2026 Jan 22;27(2):1093. doi: 10.3390/ijms27021093 (PMC12841778; doi:10.3390/ijms27021093)
Supplement: Supplementary file 1 [file ijms-27-01093-s001.zip › ijms-4051385-supplementary materials.pdf]

Supplementary Tables & Figures

Supplementary Table S1. Full electronic search strategies for all databases. This table provides complete, line-by-line search strategies used for PubMed/MEDLINE, Embase, the Cochrane Library, Scopus, and ClinicalTrials.gov, including controlled vocabulary (MeSH and Emtree terms) and free-text keywords related to psoriasis, psoriatic arthritis, antinuclear antibody (ANA) positivity, cutaneous lupus erythematosus, systemic lupus erythematosus, biologic and targeted therapies, phototherapy, and drug-induced lupus. Search limits (English language; human studies) are reported to facilitate reproducibility. Search period: database inception through 31 October 2025.

PubMed/MEDLINE (via NCBI).

Line	Search string
#1	Psoriasis / Psoriatic arthritis – ("Psoriasis"[Mesh] OR "Psoriasis"[tiab] OR psoria*[tiab] OR "Arthritis, Psoriatic"[Mesh] OR "psoriatic arthritis"[tiab] OR "PsA"[tiab])
#2	Lupus-spectrum conditions – ("Lupus Erythematosus, Cutaneous"[Mesh] OR "cutaneous lupus"[tiab] OR "cutaneous lupus erythematosus"[tiab] OR "CLE"[tiab] OR "Lupus Erythematosus, Systemic"[Mesh] OR "systemic lupus erythematosus"[tiab] OR "SLE"[tiab])
#3	Autoantibodies / ANA – ("Antinuclear Antibodies"[Mesh] OR "antinuclear antibody"[tiab] OR "antinuclear antibodies"[tiab] OR "ANA"[tiab] OR "ANA-positive"[tiab] OR "ANA positivity"[tiab])
#4	Biologic / targeted systemic therapies – ("Biological Products"[Mesh] OR "biologic*"[tiab] OR "monoclonal antibodies"[Mesh] OR "targeted therapy"[tiab] OR "Tumor Necrosis Factor-alpha/antagonists & inhibitors"[Mesh] OR "Interleukin-17/antagonists & inhibitors"[Mesh] OR "Interleukin-23/antagonists & inhibitors"[Mesh] OR "Janus Kinase Inhibitors"[Mesh] OR "Phosphodiesterase 4 Inhibitors"[Mesh] OR "TYK2"[tiab] OR deucravacitinib[tiab] OR ustekinumab[tiab] OR secukinumab[tiab] OR ixekizumab[tiab] OR brodalumab[tiab] OR guselkumab[tiab] OR risankizumab[tiab] OR tildrakizumab[tiab] OR infliximab[tiab] OR adalimumab[tiab] OR etanercept[tiab] OR certolizumab[tiab] OR golimumab[tiab] OR apremilast[tiab])
#5	Phototherapy / UV exposure – ("Phototherapy"[Mesh] OR phototherap*[tiab] OR "ultraviolet therapy"[tiab] OR "UVB"[tiab] OR "NB-UVB"[tiab] OR "narrowband UVB"[tiab] OR "PUVA"[tiab])
#6	Core disease concept – #1 AND (#2 OR #3)
#7	Core treatment concept – (#4 OR #5)
#8	Final search – #6 AND #7
#9	Limits applied – #8 AND (english[lang]) AND (humans[mesh])

Embase (via Ovid or Elsevier).

Line	Search string (Emtree and free text)
#1	'psoriasis'/exp OR psoria*:ti,ab
#2	'psoriatic arthritis'/exp OR "psoriatic arthritis":ti,ab OR 'PsA':ti,ab
#3	'cutaneous lupus erythematosus'/exp OR "cutaneous lupus":ti,ab OR "cutaneous lupus erythematosus":ti,ab OR CLE:ti,ab
#4	'systemic lupus erythematosus'/exp OR "systemic lupus erythematosus":ti,ab OR SLE:ti,ab
#5	'antinuclear antibody'/exp OR "antinuclear antibodies":ti,ab OR "antinuclear antibody":ti,ab OR ANA:ti,ab
#6	'biological therapy'/exp OR biologic*:ti,ab OR 'monoclonal antibody'/exp OR 'targeted therapy'/exp OR "targeted therapy":ti,ab
#7	Named agents – etanercept:ti,ab OR infliximab:ti,ab OR adalimumab:ti,ab OR certolizumab:ti,ab OR golimumab:ti,ab OR secukinumab:ti,ab OR ixekizumab:ti,ab OR brodalumab:ti,ab OR ustekinumab:ti,ab OR guselkumab:ti,ab OR risankizumab:ti,ab OR tildrakizumab:ti,ab OR deucravacitinib:ti,ab OR apremilast:ti,ab

Line	Search string (Emtree and free text)
#8	'phototherapy'/exp OR phototherap*:ti,ab OR 'ultraviolet therapy':ti,ab OR 'UVB':ti,ab OR 'NB-UVB':ti,ab OR 'PUVA':ti,ab
#9	(#1 OR #2) AND (#3 OR #4 OR #5)
#10	(#6 OR #7 OR #8)
#11	#9 AND #10
#12	Limits: #11 AND [english]/lim AND [humans]/lim

Cochrane Library (CENTRAL).

Line	Search string
#1	psoriasis:ti,ab,kw OR "psoriatic arthritis":ti,ab,kw OR PsA:ti,ab,kw
#2	"cutaneous lupus":ti,ab,kw OR "cutaneous lupus erythematosus":ti,ab,kw OR CLE:ti,ab,kw OR "systemic lupus erythematosus":ti,ab,kw OR SLE:ti,ab,kw
#3	"antinuclear antibody":ti,ab,kw OR "antinuclear antibodies":ti,ab,kw OR ANA:ti,ab,kw
#4	biologic*:ti,ab,kw OR "biological therapy":ti,ab,kw OR "targeted therapy":ti,ab,kw OR apremilast:ti,ab,kw OR deucravacitinib:ti,ab,kw OR ustekinumab:ti,ab,kw OR secukinumab:ti,ab,kw OR ixekizumab:ti,ab,kw OR guselkumab:ti,ab,kw OR risankizumab:ti,ab,kw OR infliximab:ti,ab,kw OR adalimumab:ti,ab,kw OR etanercept:ti,ab,kw
#5	phototherap*:ti,ab,kw OR "ultraviolet therapy":ti,ab,kw OR UVB:ti,ab,kw OR "NB-UVB":ti,ab,kw OR PUVA:ti,ab,kw
#6	(#1) AND (#2 OR #3)
#7	(#4 OR #5)
#8	#6 AND #7 (no automatic language/human filters applied; records later screened manually for English-language human studies)

Scopus (Elsevier). *Search fields: TITLE-ABS-KEY*

Line	Search string
#1	TITLE-ABS-KEY(psoriasis OR "psoriatic arthritis" OR PsA)
#2	TITLE-ABS-KEY("cutaneous lupus" OR "cutaneous lupus erythematosus" OR CLE OR "systemic lupus erythematosus" OR SLE)
#3	TITLE-ABS-KEY("antinuclear antibody" OR "antinuclear antibodies" OR ANA)
#4	TITLE-ABS-KEY(biologic* OR "biological therapy" OR "targeted therapy" OR apremilast OR deucravacitinib OR ustekinumab OR secukinumab OR ixekizumab OR guselkumab OR risankizumab OR infliximab OR adalimumab OR etanercept OR certolizumab OR golimumab)
#5	TITLE-ABS-KEY(phototherap* OR "ultraviolet therapy" OR UVB OR "NB-UVB" OR PUVA)
#6	(#1 AND (#2 OR #3)) AND (#4 OR #5)
Limits	Document type: Article OR Review; Language: English; Humans (where indexed). Final search date: 31 October 2025.

ClinicalTrials.gov. Search fields: Condition or disease; Other terms; Study type filters applied using website interface.

Field	Search entry
Condition or disease	psoriasis OR "psoriatic arthritis"
Other terms	"systemic lupus erythematosus" OR "cutaneous lupus" OR CLE OR SLE OR ANA
Study type filters	Interventional studies (clinical trials); Phase 2–4; Adult (18+).
Additional filters	Recruiting, active, or completed; English language records where available.

Field	Search entry
Final combined query (free-text view)	psoriasis OR "psoriatic arthritis" AND ("systemic lupus erythematosus" OR "cutaneous lupus" OR CLE OR SLE OR ANA) AND (biologic OR biologic* OR apremilast OR deucravacitinib OR ustekinumab OR secukinumab OR ixekizumab OR guselkumab OR risankizumab OR infliximab OR adalimumab OR etanercept OR certolizumab OR golimumab)

Supplementary Table S2. Risk of Bias Assessments for Included Studies. Summary of quality evaluations for all included studies using Cochrane Risk of Bias 2.0 for randomized trials, the Newcastle–Ottawa Scale (NOS) for observational studies, and the Murad methodological tool for case series. Ratings are presented for each study across domains of selection, comparability, outcome measurement, and reporting completeness.

Study (first author, year)	Study design	RoB tool	Selection	Comparability	Outcome measurement	Reporting completeness	Overall RoB
Pink 2010 [35]	Prospective cohort (PsO, anti-TNF, ANA)	Newcastle–Ottawa	Low	Moderate	Low	Moderate	Moderate
Pirowska 2015 [36]	Prospective cohort (PsO/PsA, anti-TNF, ANA)	Newcastle–Ottawa	Low	Moderate	Low	Moderate	Moderate
Bardazzi 2014 [37]	Prospective cohort (PsO, anti-TNF)	Newcastle–Ottawa	Low	Moderate	Moderate	Moderate	Moderate
Oter-López 2017 [38]	Retrospective cohort (PsO, anti-TNF)	Newcastle–Ottawa	Moderate	Moderate	Moderate	Moderate	Moderate
Yanaba 2016 [39]	Small prospective series (PsO, ustekinumab)	Murad tool	Moderate	High	Moderate	Moderate	Moderate–High
Miki 2019 [40]	Prospective cohort (PsO, secukinumab)	Newcastle–Ottawa	Moderate	Moderate	Low	Moderate	Moderate
Kutlu 2020 [41]	Case series (PsO, mixed biologics)	Murad tool	Moderate	High	Moderate	Moderate	Moderate–High
Sugiura 2021 [42]	Prospective cohort (PsO, ixekizumab)	Newcastle–Ottawa	Low	Moderate	Low	Moderate	Moderate
Miyazaki 2023 [43]	Case series (PsO, guselkumab)	Murad tool	Moderate	High	Moderate	Moderate	Moderate–High
Staniszewska 2025 [44]	Case series (PsO/PsA + CLE overlap)	Murad tool	Moderate	High	Moderate	Moderate	Moderate–High
García-Arpa 2019 [45]	Small case series / mixed cases (PsO/PsA + CLE)	Murad tool	Moderate	High	Moderate	Moderate	Moderate–High
De Souza 2012 [46]	Case series (TNF-induced SCLE)	Murad tool	Moderate	High	Moderate	Moderate	Moderate–High
Sachdeva 2020 [47]	Case series (TNF-induced CLE in PsO)	Murad tool	Moderate	High	Moderate	Moderate	Moderate–High
Prieto-Barrios 2017 [48]	Mixed cohort / series (PsO ± CLE/SLE)	Newcastle–Ottawa	Moderate	Moderate	Moderate	Moderate	Moderate
Zalla & Muller 1996 [49]	Retrospective cohort (PsO + photosensitive LE disorders)	Newcastle–Ottawa	Moderate	High	Moderate	Moderate	Moderate–High
Hays 1984 [50]	Case series (PsO + SLE)	Murad tool	Moderate	High	Moderate	High	High
Tselios 2017 [51]	Retrospective series (SLE with PsO)	Newcastle–Ottawa	Moderate	Moderate	Moderate	Moderate	Moderate

Study (first author, year)	Study design	RoB tool	Selection	Comparability	Outcome measurement	Reporting completeness	Overall RoB
Ali 2025 [52]	Case series (PsO/PsA + SLE on anti-TNF)	Murad tool	Moderate	High	Moderate	Moderate	Moderate–High
Walhelm 2025 [53]	Registry-based cohort (SLE with PsO)	Newcastle–Ottawa	Low–Moderate	Moderate	Moderate	Moderate	Moderate
Johnson 2005 [55]	Cohort (PsA, anti-TNF, ANA)	Newcastle–Ottawa	Moderate	Moderate	Moderate	Moderate	Moderate
Silvy 2015 [56]	Cohort (PsA, anti-TNF)	Newcastle–Ottawa	Moderate	Moderate	Moderate	Moderate	Moderate
Viana 2010 [54]	Cohort (PsA, anti-TNF, autoantibodies)	Newcastle–Ottawa	Moderate	Moderate	Moderate	Moderate	Moderate
Kara 2025 [57]	Case series (ANA+ PsA on anti-TNF)	Murad tool	Moderate	High	Moderate	Moderate	Moderate–High
Eibl 2023 [58]	Conference cohort (PsA, ANA on anti-TNF)	Murad tool (abstract only)	High	High	High	High	High
Walz LeBlanc 2020 [59]	Short case series (TNF-induced CLE in PsA)	Murad tool	High	High	Moderate	High	High
Avriel 2007 [61]	Case series (PsA + SLE)	Murad tool	Moderate	High	Moderate	Moderate	Moderate–High
Bonilla 2016 [62]	Retrospective SLE cohort (PsO/PsA incidence)	Newcastle–Ottawa	Moderate	Moderate	Moderate	Moderate	Moderate
Korkus 2021 [63]	Population case–control (PsA with SLE prevalence)	Newcastle–Ottawa	Moderate	Moderate	Moderate	Moderate	Moderate
Sato 2020 [64]	Case report (PsA + SLE on secukinumab)	Murad tool	High	High	Moderate	High	High
Venetsanopoulou 2025 [65]	Case-based series + review (PsA + SLE)	Murad tool	Moderate	High	Moderate	Moderate	Moderate–High

Supplementary Table S3. Detailed Adverse Event Profiles Across Psoriasis–Lupus Overlap Populations. Granular listing of lupus-related and psoriasis-related safety events reported across included studies, including ANA seroconversion, dsDNA induction, CLE exacerbation, SLE flares, drug-induced lupus (DIL), hydroxychloroquine-associated psoriasis flares, and patterns of interferon activation where available.

Study (first author, year)	Population / overlap phenotype	Main systemic therapy exposure	ANA seroconversion / ANA ↑	Anti-dsDNA induction	CLE worsening / induction	SLE flares / DIL	Psoriasis / PsA flares (e.g., HCQ)	IFN-related or mechanistic signals	Notes / comments
Pink 2010 [35]	Psoriasis, anti-TNF; ANA cohort	Etanercept	↑ ANA; some high-titer	Not systematically reported	None	No clinical lupus; ANA ↑ associated with TNF	None	Not assessed	Classic PsO + ANA cohort; "ANA without

Study (first author, year)	Population / overlap phenotype	Main systemic therapy exposure	ANA seroconversion / ANA ↑	Anti-dsDNA induction	CLE worsening / induction	SLE flares / DIL	Psoriasis / PsA flares (e.g., HCQ)	IFN-related or mechanistic signals	Notes / comments
						loss of response			lupus" concept
Pirowska 2015 [36]	PsO/PsA on anti-TNF	Infliximab, adalimumab	ANA seroconversion 20–30%	Occasional dsDNA ↑	None	No DIL; no SLE	None	Not assessed	TNF-induced autoimmunity without lupus
Bardazzi 2014 [37]	PsO on anti-TNF	Various anti-TNF	Frequent ANA ↑	Rare dsDNA ↑	None	Possible mild lupus-like signs	None	Not assessed	Autoantibody induction under TNF blockade
Oter-López 2017 [38]	PsO moderate–severe	Anti-TNF	Frequent ANA ↑	dsDNA ↑ in subset	Occasional CLE rash	Several probable DIL/SLE (all resolved after withdrawal)	None	Not assessed	One of the strongest DIL datasets (~6–15%)
Yanaba 2016 [39]	Japanese PsO	Ustekinumab	Mild ANA ↑	Not reported	None	None	None	Not assessed	IL-12/23 blockade: ANA-neutral
Miki 2019 [40]	PsO	Secukinumab	ANA stable	dsDNA not tracked	None	No SLE/DIL	None	Not assessed	IL-17 ANA-neutral; later balanced by CLE case reports
Kutlu 2020 [41]	PsO (mixed biologics)	Anti-TNF, ustekinumab	Mild ANA ↑	Not reported	None	No lupus	None	Not assessed	Benign ANA changes in non-TNF agents
Sugiura 2021 [42]	PsO	Ixekizumab	ANA stable	Not reported	None	None	None	Not assessed	IL-17 ANA-stable
Miyazaki 2023 [43]	PsO	Guselkumab	Some ANA ↑	Not reported	None	None	None	Not assessed	No CLE/SLE despite ANA ↑
Staniszewska 2025 [44]	PsO ± PsA + CLE	Various biologics	Baseline ANA+ common	dsDNA limited	CLE worsened by TNF; improved after IL-23	No SLE flares	None	CLE = IFN-high	Class-specific CLE risk summary
García-Arpa 2019 [45]	PsO/PsA + CLE	Anti-TNF	ANA+	Not reported	Clear TNF-induced CLE	No systemic SLE	None	Not assessed	Classic TNF-CLE example
De Souza 2012 [46]	PsO + SCLE	Anti-TNF	ANA & anti-Ro/La ↑	Variable	SCLE induction	Cutaneous-only lupus	None	Not assessed	TNF → SCLE

Study (first author, year)	Population / overlap phenotype	Main systemic therapy exposure	ANA seroconversion / ANA ↑	Anti-dsDNA induction	CLE worsening / induction	SLE flares / DIL	Psoriasis / PsA flares (e.g., HCQ)	IFN-related or mechanistic signals	Notes / comments
									pathophysiology
Sachdeva 2020 [47]	PsO + TNF-induced CLE	Anti-TNF	ANA ↑	dsDNA ↑ occasionally	Multiple SCLE/DLE	Some DIL (resolved after withdrawal)	None	Not assessed	Strong CLE/DIL association
Prieto-Barrios 2017 [48]	PsO ± CLE/SLE on biologics	Etanercept, adalimumab	ANA+ common	Not systematic	CLE flares in CLE pts	SLE flares in SLE pts	None	Not assessed	Mixed PsO–CLE–SLE cohort showing TNF risks
Zalla & Muller 1996 [49]	PsO + photosensitive LE	Non-biologic	Frequent ANA+	dsDNA sometimes ↑	CLE/photosensitive LE	Occasional SLE flares	None	Not assessed	Pre-biologic coexistence evidence
Hays 1984 [50]	PsO + SLE	Conventional therapy	ANA/dsDNA high	Present	No biologic-induced CLE	SLE flares disease-driven	None	Not assessed	Very early PsO + SLE documentation
Tselios 2017 [51]	SLE with PsO	SLE therapies	ANA/dsDNA per SLE	Present	CLE common	SLE flares by baseline	Psoriasis flares with HCQ	Not assessed	Key HCQ → psoriasis flare proof
Ali 2025 [52]	PsO/PsA + SLE on TNF	Anti-TNF	ANA high baseline	dsDNA+ common	CLE rashes in some	Clear TNF-related SLE flares	Some PsO instability	Not assessed	TNF → SLE flares in overlap
Walhelm 2025 [53]	Registry SLE + PsO	Mixed biologics	ANA/dsDNA per SLE	Present	CLE frequent	Flares mainly with TNF	HCQ → psoriasis flares	Not measured	Registry-level: TNF worst; IL-23 safest
Johnson 2005 [55]	PsA on TNF	Anti-TNF	ANA ↑	Rare dsDNA ↑	None	No DIL	None	Not assessed	ANA-only without lupus
Silvy 2015 [56]	PsA	Anti-TNF	ANA ↑	dsDNA rare	None	No DIL	None	Not assessed	ANA ≠ clinical lupus
Viana 2010 [54]	PsA	Anti-TNF	ANA ↑; other autoAbs ↑	Some dsDNA ↑	None	Rare lupus-like	None	Not assessed	"Serologic autoimmunity without lupus"
Kara 2025 [57]	ANA+ PsA	Anti-TNF	Mild ANA ↑	Not reported	None	No DIL	None	Not assessed	Small series; limited events

Study (first author, year)	Population / overlap phenotype	Main systemic therapy exposure	ANA seroconversion / ANA ↑	Anti-dsDNA induction	CLE worsening / induction	SLE flares / DIL	Psoriasis / PsA flares (e.g., HCQ)	IFN-related or mechanistic signals	Notes / comments
Eibl 2023 [58]	PsA + ANA (abstract)	Anti-TNF	ANA profiles described	Not detailed	Not reported	Unclear	Not reported	Not assessed	Very limited data
Walz LeBlanc 2020 [59]	PsA + CLE	Anti-TNF	ANA+; dsDNA sometimes ↑	Present	CLE induction	Lupus-like	None	Not assessed	Illustrative TNF-CLE example
Avriel 2007 [61]	PsA + SLE	Non-biologic	ANA/dsDNA +	Present	CLE variable	SLE flares disease-driven	None	Not assessed	Early PsA+SLE
Bonilla 2016 [62]	SLE cohort w/ PsO/PsA prevalence	Mixed	ANA/dsDNA per SLE	Present	CLE common	SLE flares baseline	HCQ → psoriasis flares	Not assessed	Shows PsO/PsA prevalence in SLE
Korkus 2021 [63]	PsA with SLE prevalence	Mixed therapies	ANA/dsDNA per SLE	Present	CLE sometimes	SLE flares baseline	None	Not assessed	Epidemiologic support
Sato 2020 [64]	PsA + SLE on IL-17i	Secukinumab	ANA per SLE	Present	No CLE worsening	SLE stable	None	Not assessed	IL-17 safe in SLE (no CLE)
Venetsanopoulou 2025 [65]	PsA + SLE	Various	ANA/dsDNA per SLE	Present	CLE variable	SLE flares baseline	None	Not assessed	Case-based review

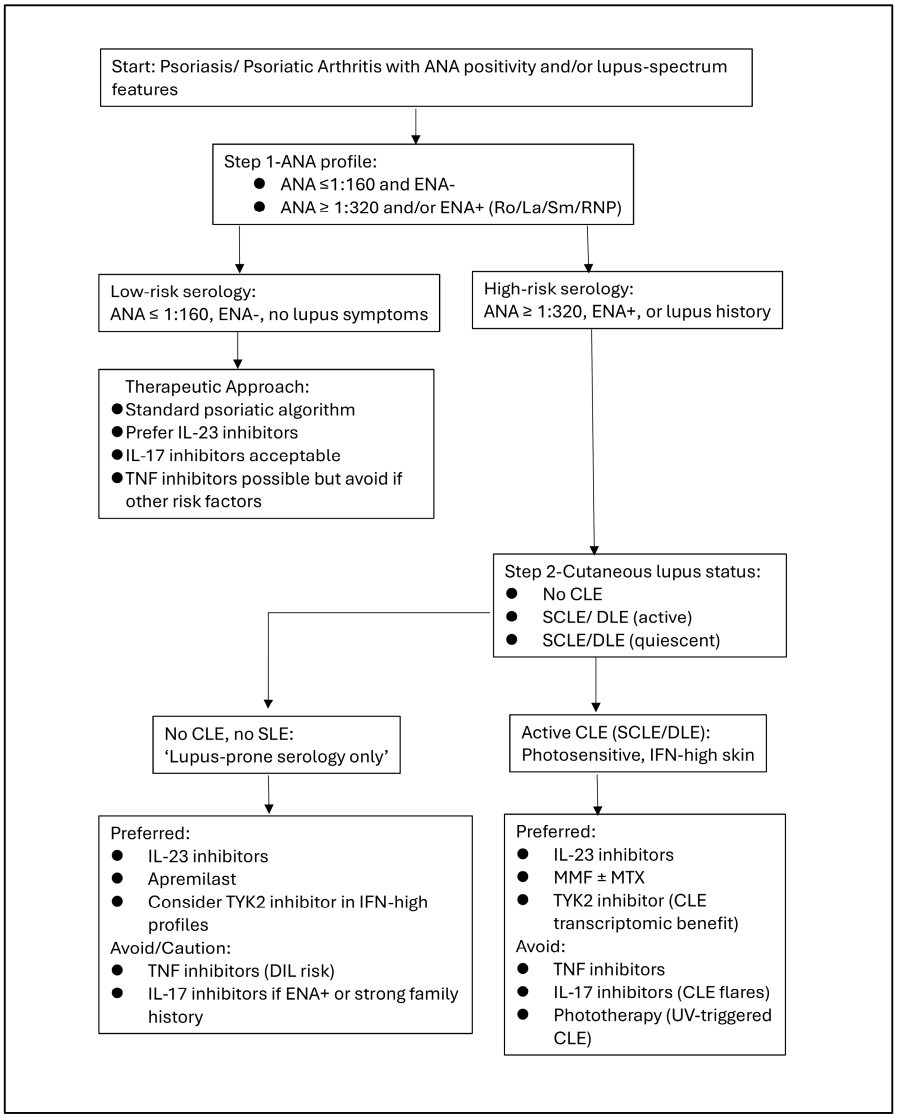

Supplementary Figure S1. Extended clinical framework for lupus and ANA risk stratification in psoriatic disease. This stepwise decision framework expands on Figure 2 and illustrates a structured approach to stratifying patients with psoriasis or psoriatic arthritis according to serologic and clinical lupus risk.

In Step 1, patients are stratified based on antinuclear antibody (ANA) titer (≤1:160 versus ≥1:320) and extractable nuclear antigen (ENA) status, distinguishing lower-risk serologic profiles from higher-risk or lupus-prone patterns.

In Step 2, cutaneous lupus erythematosus (CLE) status and subtype—subacute CLE (SCLE) versus discoid lupus erythematosus (DLE), and active versus quiescent disease—are incorporated. In this context, the framework highlights systemic therapies more frequently associated with favorable safety profiles in CLE-dominant or interferon-high phenotypes (e.g., IL-23 inhibitors, mycophenolate mofetil, methotrexate, and TYK2 inhibition), while indicating therapies more often associated with lupus-related or cutaneous safety concerns (e.g., TNF-α inhibitors, IL-17 inhibitors, and phototherapy).

This framework is intended to support phenotype-informed and mechanism-aware interpretation rather than to provide prescriptive treatment recommendations.
